# An anti-CD19/CTLA-4 switch improves efficacy and selectivity of CAR T cells targeting CD80/86-upregulated DLBCL

**DOI:** 10.1016/j.xcrm.2024.101421

**Published:** 2024-02-09

**Authors:** Lars Fabian Prinz, Tobias Riet, Daniel Felix Neureuther, Simon Lennartz, Danuta Chrobok, Hanna Hübbe, Gregor Uhl, Nicole Riet, Petra Hofmann, Marianna Hösel, Adrian Georg Simon, Luis Tetenborg, Paul Segbers, Joji Shimono, Philipp Gödel, Hyatt Balke-Want, Ruth Flümann, Gero Knittel, Hans Christian Reinhardt, Christoph Scheid, Reinhard Büttner, Björn Chapuy, Roland Tillmann Ullrich, Michael Hallek, Markus Martin Chmielewski

**Affiliations:** 1 Department I of Internal Medicine, University Hospital Cologne, 50937 Cologne, Germany; 2 Center for Molecular Medicine Cologne (CMMC), 50931 Cologne, Germany; 3 Heidelberg University, 69117 Heidelberg, Germany; 4 Institute of Pathology, University Hospital Cologne, 50937 Cologne, Germany; 5 Department of Hematology, Oncology and Tumorimmunology, Charité University Medical Center Berlin, Benjamin Franklin Campus, 12203 Berlin, Germany; 6 Cologne Excellence Cluster on Cellular Stress Response in Aging-Associated Diseases (CECAD), University of Cologne, 50931 Cologne, Germany; 7 Mildred Scheel School of Oncology Aachen Bonn Cologne Düsseldorf (MSSO ABCD), Faculty of Medicine and University Hospital of Cologne, Cologne, Germany; 8 Max Planck Institute for Biology of Ageing, Joseph-Stelzmann-Str. 9b, 50931 Cologne, Germany; 9 University Hospital Essen, Department of Hematology and Stem Cell Transplantation, West German Cancer Center, German Cancer Consortium Partner Site Essen, Center for Molecular Biotechnology, Hufelandstr. 55, 45147 Essen, Germany; 10 Stanford Center for Cancer Cell Therapy, Stanford Cancer Institute, Stanford University, Stanford, CA, USA

**Keywords:** CAR T cells, lymphoma, DLBCL, FL, checkpoint ligand, chimeric checkpoint receptor, neurotoxicity, CD19, CD80, CD86

## Abstract

Chimeric antigen receptor T cell (CAR T) therapy is a potent treatment for relapsed/refractory (r/r) B cell lymphomas but provides lasting remissions in only ∼40% of patients and is associated with serious adverse events. We identify an upregulation of CD80 and/or CD86 in tumor tissue of (r/r) diffuse large B cell lymphoma (DLBCL) patients treated with tisagenlecleucel. This finding leads to the development of the CAR/CCR (chimeric checkpoint receptor) design, which consists of a CD19-specific first-generation CAR co-expressed with a recombinant CTLA-4-linked receptor with a 4-1BB co-stimulatory domain. CAR/CCR T cells demonstrate superior efficacy in xenograft mouse models compared with CAR T cells, superior long-term activity, and superior selectivity in *in vitro* assays with non-malignant CD19^+^ cells. In addition, immunocompetent mice show an intact CD80^−^CD19^+^ B cell population after CAR/CCR T cell treatment. The results reveal the CAR/CCR design as a promising strategy for further translational study.

## Highlights

• Some DLBCL patients exhibit elevated CD80/86 expression pre- and post-CAR T therapy
• Switch CAR/CCR T cells show higher efficacy than conventional CAR T cells *in vivo*
• The switch CAR/CCR design reduces on-target off-tumor activity *in vitro* and *in vivo*

## Graphical abstract

**Figure:**
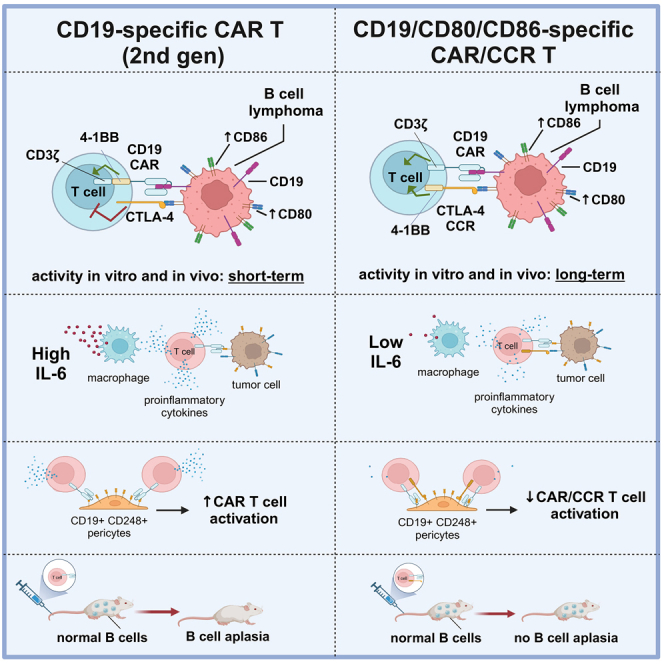

## Teaser

Prinz et al. present an “AND” switch CAR T cell construct that fully activates only when it binds to CD19 and CD80/CD86 found on lymphoma cells. Compared with CAR T cells targeting only CD19, they reveal improved anti-tumor activity while sparing healthy CD19-expressing cells, like non-malignant B cells and metaneural pericytes.

## Introduction

The treatment of B cell malignancies with T cells engineered to express CD19-specific CAR constructs remains the most impactful application of CAR therapy. Despite the unprecedented efficacy of anti-CD19 CAR T cell therapy in relapsed or refractory diffuse large B cell lymphoma (DLBCL), long lasting remissions are observed in only about 40% of patients.^1^^,^^2^^,^^3^^,^^4^^,^^5^^,^^6^ Since the first report in 2010, the US Food and Drug Administration has approved four anti-CD19 products in non-Hodgkin lymphoma (NHL), all of which belong to the second-generation (2^nd^ Gen) CD19 CAR T cell product family.^7^^,^^8^ These drugs have been clinically successful despite considerable toxicity, including cytokine release syndrome (CRS) and immune effector cell-associated neurotoxicity syndrome (ICANS). Even though the clinical management of CRS and ICANS has improved with the widespread use of tocilizumab and corticosteroids, immune-mediated toxicities represent significant challenges to patient safety and ease of treatment.^9^ The detailed mechanisms of CAR T cell tumoricidal activity and toxicity remain poorly understood, due in part to a lack of information on the interactions in the tumor microenvironment (TME) after product infusion.^10^ Recently, Parker et al. identified a small population of brain mural cells expressing CD19 that might serve as potential off-tumor targets for CAR T immunotherapies and could play a role in the pathophysiology of ICANS,^11^ next to or on top of cytokine-induced dysfunction of the blood-brain barrier and transmigration of inflammatory cells.^12^ B cell aplasia and resultant hypogammaglobulinemia are other common consequences of CD19 CAR T cell-based immunotherapy, putting patients at risk for infectious complications.^13^^,^^14^^,^^15^^,^^16^ Besides unsolved safety issues, there is an enormous need for increased efficacy of CAR T cell-based immunotherapy in B cell lymphoma. Although not all resistance mechanisms are understood, there is increasing evidence that distinct control mechanisms within the TME can sustainably dampen CAR T cell activity, despite unaltered expression of the CD19 target antigen on malignant B cells.^17^^,^^18^ These include prominent immune checkpoint receptors such as PD-1 and CTLA-4, which are also implicated in the immune escape of tumors not treated with CARs.^19^^,^^20^ The use of immune-checkpoint inhibitory antibodies targeting these receptors or their ligands has recently revolutionized the treatment of several tumor entities,^21^^,^^22^ but only a modest antitumor activity was observed in patients suffering from DLBCL on PD-1 and CTLA-4 blockade therapy.^23^^,^^24^ There are promising approaches that combine CAR T cell and immune checkpoint treatment to support the reactivation of exhausted CAR T cells.^25^^,^^26^^,^^27^^,^^28^^,^^29^ Notably, such a therapy is non-specific to CAR T cells and might also contribute to the reactivation of autoreactive T cells leading to severe autoimmune responses in some patients.^30^ In this work we reveal that the expression of CD80 and/or CD86 is upregulated in most DLBCL patients receiving CAR T cell therapy and that a co-targeting CAR/CCR (chimeric checkpoint receptor) design increases both efficacy and safety of the CD19-specific CAR T cells compared with T cells expressing a 2^nd^ Gen CAR T construct.

## Results

### Immune checkpoint ligands CD80 and CD86 are expressed in most tumor biopsies derived from DLBCL patients after receiving CAR (2^nd^ Gen) T cell products

CD80 and CD86 (B7-1 and B7-2, respectively) are ligands for both immunostimulatory CD28 and immunosuppressive CTLA-4 receptors expressed on T cells (CD4/CD8/T_reg_). However, CTLA-4 harbors a higher affinity for CD80 and for CD86 than CD28, thereby displacing CD28 when expressed.^31^ We first investigated whether CAR T cell therapy based on 2^nd^ Gen CARs specific for CD19 (Tisagenleceucel, Novartis), CD20 (Miltenyi), and bispecific for CD20 and CD19 (Miltenyi) has an impact on CD80/86 expression on B cell lymphoma tissue. Therefore, we analyzed matched tumor biopsies derived from seven patients with aggressive B-NHL (six DLBCL, one follicular lymphoma after transformation into an aggressive lymphoma [tFL]) and one follicular lymphoma (FL) patient before and after CAR T cell treatment by immunohistochemistry analyses for the expression of both ligands, CD80 and CD86.

**Figure 1:**
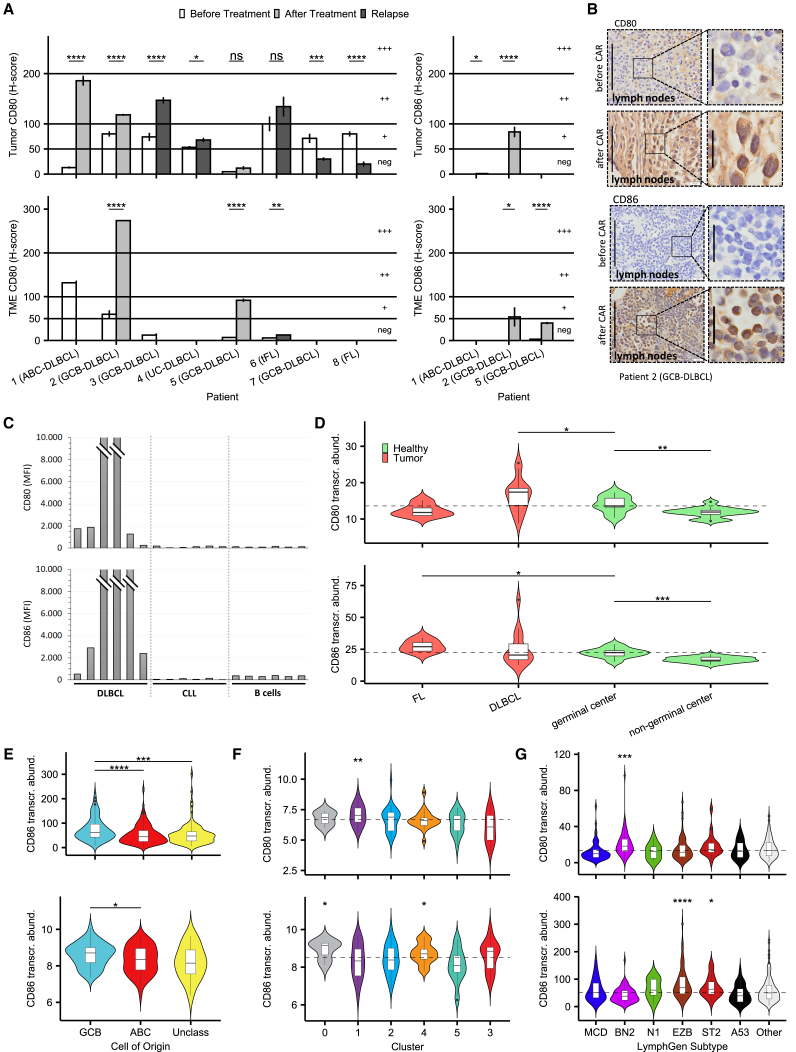
Immune checkpoint ligands CD80 and CD86 are expressed in most tumor biopsies derived from DLBCL patients after receiving CAR (2^nd^ Gen) T cells (A) Evaluation of CD80/86 expression in lymphoma slides before and after treatment or at relapse as a mean H score, separated into tumor and tumor microenvironment cells where possible. See also Figures S1A‒S1C. Bar plots represent mean ± SEM of five high-power fields per slide. Statistical significance levels were determined by using a non-adjusted t test and reported according to p values (thresholds below). (B) Representative optical field section of DLBCL patient lymph node slides stained to show CD80/CD86 expression before and after treatment with tisagenlecleucel. Scale bars, 100 μm (original) and 20 μm (zoomed images). (C) Antigen expression of CD80 and CD86 in singularized DLBCL biopsy samples (CD19^+^/CD5^–^ gated) and peripheral blood samples of CLL (CD19^+^/CD5^+^ gated) and healthy donors (CD19^+^/CD20^+^ gated) measured by flow cytometry and plotted in comparison. Bar plots represent mean fluorescence intensity of each sample. (D) Comparison of CD80/CD86 transcript abundance across different cell types and lymphoma entities in the Brune et al. set.^32^ The horizontal line marks the median abundance of CD80/86 in healthy germinal center B cells. (E) CD86 transcriptional abundance across COO classification, upper plot from the Schmitz et al. set^33^ and lower plot from the Chapuy et al. set.^34^ (F and G) CD80/CD86 transcriptional abundance across genetic subtype clusters in the Chapuy et al. set and across revised LymphGen subtypes in the Schmitz et al. set. Horizontal line marks median abundance of all subtypes, which also serves as the reference group for individual group statistical testing. Statistical significance in transcriptomic data was evaluated using a one-sided unpaired non-adjusted Mann-Whitney U test and significance levels reported according to p values: ∗p ≤ 0.05; ∗∗p ≤ 0.01; ∗∗∗p ≤ 0.001; ∗∗∗∗p ≤ 0.0001.

Based on H-score analysis and differentiation of TME cells by a pathologist, we revealed an increased and notable expression of CD80 and CD86 on tumor and/or TME cells in five patients, a maintained notable expression in one patient, and a decrease into negative expression in two patients following CAR T cell immunotherapy (Figures 1A and 1B). The decrease was observed in patient 7, unique in this study for being treated with an anti-CD20 CAR, and patient 8, unique for being classified as a follicular lymphoma patient (Figures S1A‒S1C). We also analyzed CD80/CD86 expression on singularized B cells (CD19^+^/CD5^–^ gated) from DLBCL biopsies. As demonstrated in Figure 1C, CD80 and/or CD86 are expressed on the surface of these B cells from most DLBCL patients, although we could not differentiate between lymphoma and other B cells. These patients had not yet received CAR T cell therapy and were compared with peripheral blood samples from CLL patients (CD19^+^/CD5^+^ gated) and from healthy donors (CD19^+^/CD20^+^ gated), both of which showed no notable surface expression of CD80/CD86. These results confirm our hypothesis that, in most DLBCL patients, regardless of CAR T cell treatment, one or even both ligands for CTLA-4 are already upregulated on the surface of lymph node B and lymphoma cells.

We also confirmed this increased expression in published transcriptomic datasets. Using a publicly available transcriptional human dataset composed of sorted normal B cells and B cell lymphomas,^32^ we identified a significant increase in CD80 and CD86 transcript abundance in non-malignant germinal center B cells (centroblasts, centrocytes) compared with non-germinal center B cells (naive B cells, memory B cells, plasma cells). DLBCL (DLBCL-NOS [not otherwise specified] and TCHRBCL [T cell/histiocyte-rich B cell lymphoma]) and FL samples showed an even higher expression of CD80 and CD86, respectively (Figure 1D). Next, we analyzed the transcript abundance of CD80 and CD86 in two datasets of primary DLBCL samples reported by Chapuy et al.^34^ and Schmitz et al.,^33^ revealing an increased expression of CD86 in the transcriptional germinal center B cell (GCB)-DLBCL-type compared with the activated B cell type DLBCLs (Figures 1E and S1D). Consistent with this, CD86 was significantly higher expressed in the GCB-enriched genetic subtypes C4^34^ and the revised LymphGen subtypes^35^ EZB and ST2. CD80 was increased in the C1/BN2 subtypes (Figures 1F and 1G). In summary, these data suggest an increased expression of CD80/CD86 in aggressive B cell lymphomas beyond its physiological upregulation during the germinal center reaction.

### Design, expression, and activity of CD19/CD80/CD86-specific CAR/CCR T cells

**Figure 2:**
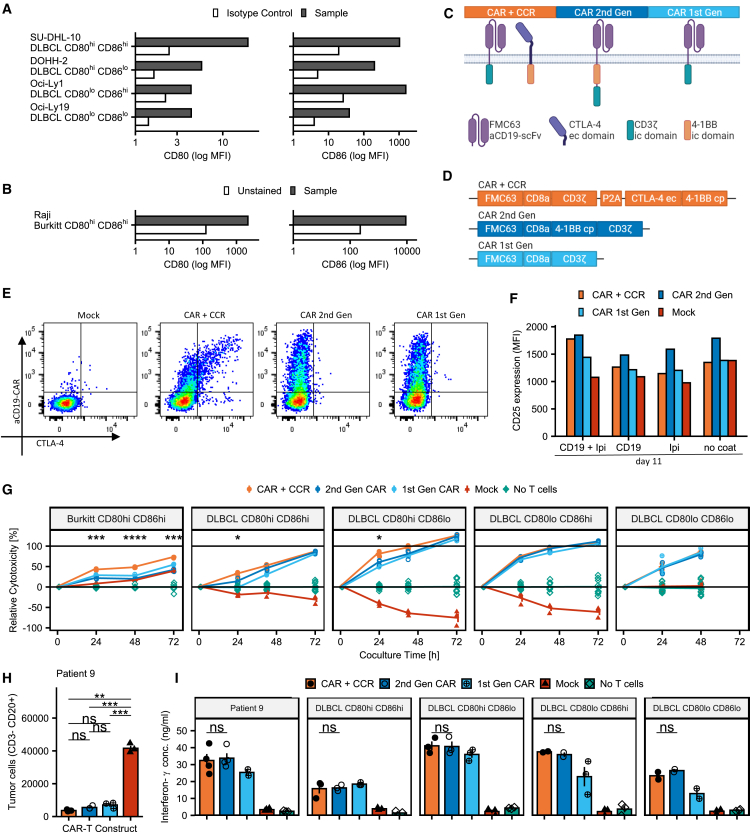
Design, expression, and activity of CD19/CD80/86-specific CAR T cells (A) Expression profiles of CD80 and CD86 in DLBCL cell lines used for *in vitro* experiments in this paper. See also Figure S2A. (B) Expression profiles of CD80 and CD86 in the Raji cell line used for *in vitro* and *in vivo* experiments. (C) Stylized representation of receptors used in this study. (D) Stylized representation of transduction vectors used in this study. (E) Representative FACS plots showing expression of anti-CD19 CAR and CTLA-4 receptors on transduced PBMCs. See also Figures S2B and S2C. (F) Expression of CD25 on CAR (+CCR) T cells and untransduced cells stimulated with CD19 and/or Ipilimumab 11 days after first activation. Bar plots represent mean fluorescence intensity of pooled (n = 2) samples. (G) Cytotoxicity of CAR/CCR and CAR T cells in co-culture with Burkitt and DLBCL lymphoma cell lines relative to controls without T cells. Significance levels derived from t tests comparing CAR/CCR with CAR (2^nd^ Gen), unmarked comparisons are not significant. Line plots represent mean ± SEM, n = 3 for constructs and n = 12 for no T cell control. (H) Depletion of patient-derived tumor cells in co-culture assay evaluated using FACS and comparing CAR/CCR T cells with CAR T cells, with both showing significant cell depletion compared with Mock. Bar plots represent mean ± SEM, n = 3. (I) Interferon-γ secretion of CAR/CCR and control CAR T cells in co-culture with tumor cell lines and patient tumor cells. Bar plots represent mean ± SEM, n = 3/4). Statistical significance was evaluated using a two-sided unpaired non-adjusted t test with significance levels reported according to p values: ∗p ≤ 0.05; ∗∗p ≤ 0.01; ∗∗∗p ≤ 0.001; ∗∗∗∗p ≤ 0.0001.

To establish a range of target cells for our experiments, we analyzed the surface expression of CD80 and CD86 on four DLBCL and one Burkitt lymphoma cell line (Figures 2A, 2B, and S2A). We classified four established DLBCL cell lines with differential expression of CD80 and CD86 (high/high, high/low, low/high, low/low) for *in vitro* trials and the CD80^high^/CD86^high^ Raji cell line for *in vivo* experiments (Figures 2A and 2B). None of the DLBCL lines used in our *in vitro* studies was completely negative for expression of either CD80 or CD86.

Our findings regarding the enhanced expression of CD80/86 ligands specific for CTLA-4 on the surface of tumor cells from DLBCL patients led us to design a CAR format for CD19/CD80/CD86 expressing B cell lymphomas, to not only improve the efficacy but also the safety of CAR T cell treatment in B cell lymphoma patients. Accordingly, we designed a CAR/CCR concept composed of two surface receptors specific for CD19 and CD80/86 (Figures 2C and 2D). While the CAR construct is based on 1^st^ Gen CARs and consists of a CD19-specific binding domain fused to the CD3ζ signaling unit, the co-expressed CCR construct consists of an extracellular CTLA-4 domain fused to a 4-1BB co-stimulatory unit. Since 1^st^ Gen CARs lack a co-activating signal, the co-stimulatory 4-1BB unit integrated in the CCR construct should act as a switch that, upon binding to one of its ligands, provides the signal to fully activate the CD19-redirected CAR/CCR T cells. We thereby intend to increase the selectivity using our CAR/CCR T cell concept aiming at preventing elimination of CD19-positive but CD80/86-negative cells such as some non-malignant B cell populations or CD19-expressing brain mural cells.^11^ For the use in *in vitro* and *in vivo* experiments described in this study, human T cells were genetically engineered via retroviral transduction for stable surface expression of CAR/CCR, CAR (2^nd^ Gen), and CAR (1^st^ Gen) receptors, which was confirmed by flow cytometry using CD19-CAR- and human-CTLA-4-specific antibodies (Figures 2E and S2B). Surface expression of recombinant CTLA-4 was only detected on T cells genetically modified with the CAR/CCR construct but not with CAR (1^st^ Gen), CAR (2^nd^ Gen), or mock transduced T cells. To prevent a false-positive interpretation of CCR expression by staining of endogenous CTLA-4 on CAR/CCR-redirected T cells, we performed the same flow cytometry analysis with transfected HEK293T cells and demonstrated the expression of the CTLA-4 CCR exclusively on the CAR/CCR transfected cells (Figure S2C). This also means that the recombinant co-stimulatory CTLA-4 far outnumbers the inhibitory endogenous CTLA-4 on the surface our CAR/CCR T cells. Differentiation and checkpoint receptor expression was also investigated, with not notable differences between constructs (Figures S2E and S2F).

To demonstrate and compare the biological activity of the 4-1BB co-stimulatory domain, CAR/CCR, CAR, and mock T cells were stimulated using ipilimumab (CTLA-4-specific antibody) and/or anti-mouse Fab (CAR-specific antibody) and investigated by flow cytometry for CD25 expression on CD8^+^ cells as a marker of 4-1BB activity in activated T cells. This method was established by Oh et al. with murine T cells.^36^ As expected, CAR/CCR T cells required simultaneous CAR- and CCR-mediated stimulation for upregulation of CD25 expression, properly demonstrating the interaction of the CAR and CCR within the switch construct, while 1^st^ Gen CAR T cells showed no notable increase of CD25 compared with mock and controls without stimulating antibodies due to their lack of a recombinant 4-1BB domain (Figure 2F). Interestingly, CD25 expression on 2^nd^ Gen CAR T cells was increased even without stimulating antibodies, suggesting some tonic activity. We also confirmed that our CCR is indeed able to bind to CD80 and CD86 by exposing transfected cells to recombinant and Fc-tagged CD80 and CD86 proteins and detecting these proteins with a secondary antibody (Figure S4E).

### T cells genetically modified to express CAR/CCR constructs effectively eliminate primary DLBCL cells and DLBCL and Burkitt lymphoma cell lines *in vitro*

To characterize the cytolytic effect of the CAR/CCR T cell construct on CD19-expressing tumor cells with different expression levels of both CD80 and CD86 we prepared a range of *in vitro* co-culture experiments. Co-cultures of CAR or CAR/CCR T cells with primary patient-derived B cell lymphoma cells showed strong antitumor activity for all constructs investigated (Figure 2H). Utilizing the HIDEX Sense microplate reader platform, we assessed cytolytic activity of CAR/CCR-redirected T cells in co-culture with DLBCL and Burkitt B cell lymphoma cell lines (Figure 2A) genetically modified to stably express marker proteins GFP or tdTomato (Figure S2D). As demonstrated in Figure 2G, CAR/CCR-redirected T cells mediated effective cytolytic activity on Burkitt CD19^+^CD80^high^CD86^high^ cells and were significantly more effective in elimination of target cells than T cells equipped with CD19-specific CAR constructs of 1^st^ Gen or 2^nd^ Gen. Notably, cytotoxicity mediated by CAR/CCR T cells was also significantly enhanced at 24 h in case of CD19^+^CD80^high^CD86^high^ DLBCL cell line SU-DHL-10 compared with CAR (2^nd^ Gen) and CAR (1^st^ Gen) T cells. In further cytotoxic *in vitro* studies performed with CD19^+^ DLBCL cell lines displaying CD80^low^CD86^high^, CD80^high^CD86^low^, or even CD80^low^CD86^low^ expression levels, CAR/CCR T cells were at least as effective as T cells genetically engineered to express the CD19-specific CAR constructs of the 1^st^ Gen or 2^nd^ Gen.

We further analyzed the supernatants from performed co-culture assays for the presence of the pro-inflammatory cytokine IFN-γ released by CAR/CCR and CAR-redirected CD19-specific T cells. In addition to the malignant B cells of the DLBCL cell lines used before, we also used primary CD19^+^CD80^high^CD86^high^ B lymphoma cells from DLBCL patient 9 as target cells (Figure 2H). As demonstrated in Figure 2I, CAR/CCR-redirected T cells were activated to release IFN-γ by used target cell lines and primary DLBCL tumor cells. CAR/CCR-directed T cells were equally activated to IFN-γ secretion compared with T cells expressing a 2^nd^ Gen CAR construct. T cells grafted with CD19-specific CAR construct of the 1^st^ Gen were also activated to release IFN-γ but showed a consistently weaker activation to release IFN-γ, except for the co-culture assay performed with CD19^+^CD80^high^CD86^high^ target cells of the DLBCL line SU-DHL10.

**Figure 3:**
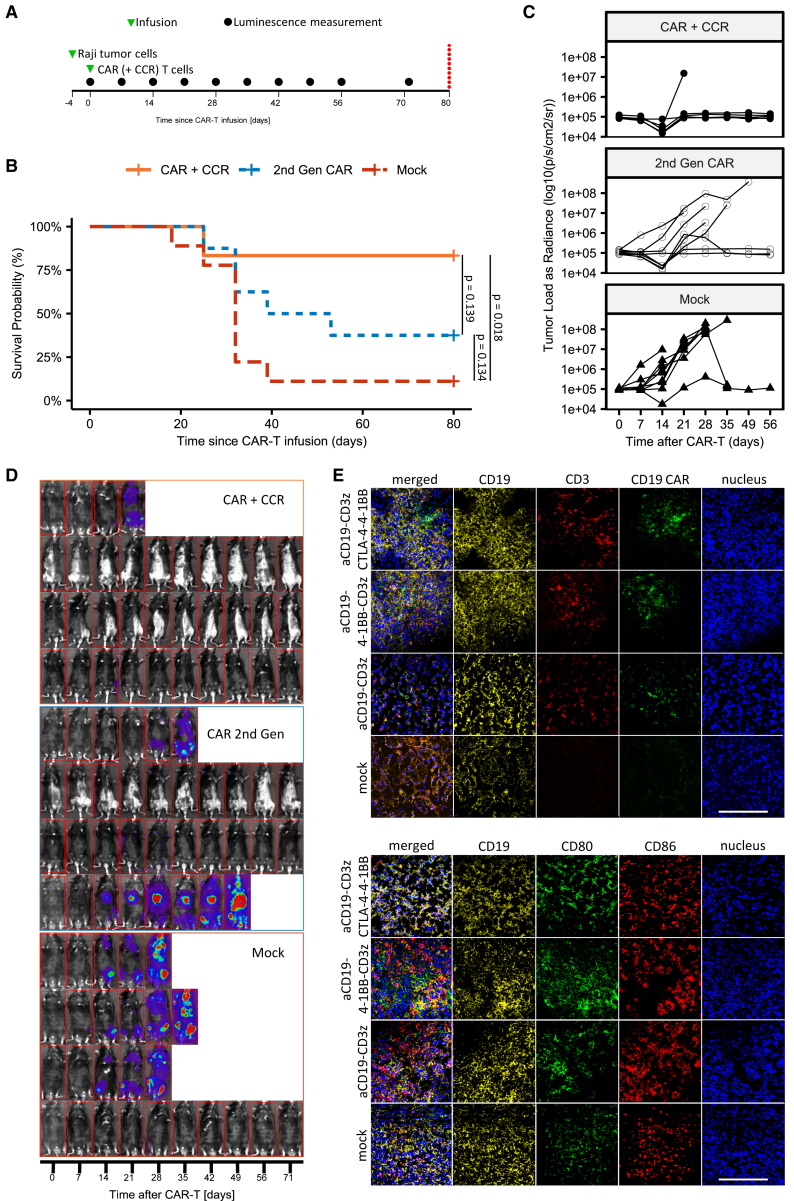
CAR/CCR T cells improve response rates and survival time of mice in xenograft B cell lymphoma model (A) Timeline of injections and luminescence measurements in Raji lymphoma xenograft mouse trial in Rag2^tm1.1Flv^ Il2rg^tm1.1Flv^ (Rag2^–^ γc^–^) mice evaluating first-line efficacy of CAR T constructs (n = 25). (B) Kaplan-Meier plot of mouse trial. Significance derived from pairwise log-rank test, p values reported. (C) Luminescence plot describing tumor burden. (D) Representative selection of mice pictures with luminescence overlay describing tumor burden. (E) Representative optical fields of spleen slides from Rag2^tm1.1Flv^ Il2rg^tm1.1Flv^ (Rag2- γc-) mice in a preliminary mouse trial. Scale bars, 100 μm.

To demonstrate the universality of our CAR/CCR approach, we performed similar *in vitro* experiments with CAR (2^nd^ Gen) and CAR/CCR T cells in which the anti-CD19 scFv binding domain of the CAR construct was replaced with a single chain specific for CD20 (Figure S3B). When co-cultured with human SU-DHL-10 (DLBCL) cells expressing CD20 (Figure S3A), CD20-specific CAR (2^nd^ Gen) and CD20/CD80/CD86-specific CAR/CCR T cells showed specific activation and cytolytic activity (Figure S3D). In an additional approach, we replaced only the extracellular CTLA-4 binding domain in the CCR with a CD86-specific scFv and left the primary CD19 binding domain unaltered (Figure S3C). After co-culture with cells of the same target cell line expressing CD19, CD80, and CD86, we observed CD19CAR/CD86CCR-specific co-activation of the redirected T cells, both in terms of antigen-specific elimination and IFN-γ release (Figure 3D). Thus, we show that the proposed CAR/CCR strategy works properly even after replacing binding domains in the CAR or CCR construct.

### CAR/CCR T cells improve response rates and survival period of mice in a xenograft B cell lymphoma model

To explore the *in vivo* anti-tumor activity of CAR/CCR T cells in comparison with CAR (2^nd^ Gen) constructs, we intravenously injected 5 × 10^4^ CD19^+^CD80^high^CD86^high^ cells of the Burkitt B cell lymphoma cell line Raji on day −4 into Rag2^–^ γc^–^ immunocompromised mice (Figure 3A). To monitor tumor progression by bioluminescence imaging we genetically modified Raji cells to express firefly Luciferase. On day 0, the bioluminescence signal in mice was recorded and mice were divided into three groups (n = 6/8/9) that were intravenously injected with 8 × 10^6^ CD19CAR/CTLA-4CCR, CD19CAR (2^nd^ Gen), or T cells (not transduced), respectively (Figure 3A). Treatment with CAR/CCR-redirected T cells resulted in B cell lymphoma clearance and complete remission in 5/6 mice until day 71, but only in 3/8 mice treated with the CAR (2^nd^ Gen) (Figures 3B–3D). As expected, the survival probability in the group of mice treated with control T cells (not transduced) as a control was significantly lower than in groups of mice that were treated with CAR/CCR T cells. Comparisons between CAR (2^nd^ Gen) and CAR/CCR groups and between CAR (2^nd^ Gen) and control T cell (not transduced) groups showed no significant differences.

Since CAR T cell persistence in tumor tissue can significantly contribute to an enhanced anti-tumor response, we investigated tumor infiltration by CD19-specific T cells after adoptive cell therapy using CAR/CCR, CAR (2^nd^ Gen), CAR (1^st^ Gen), or untransduced T cells in Raji Burkitt lymphoma-bearing mice. The presence of CD19CAR-positive T cells in tumor tissues was recorded through immune histology by staining with fluorochrome-labelled antibodies specific for CD19, CD3, and CD19scFv (FMC63) antigen epitopes. Immunohistochemistry analyses performed on tumor tissue samples revealed persistence of all CAR and CAR/CCR T cells in targeted CD19^+^ tumor tissues. Crucially, immunohistological analyses also revealed no loss of CD19, CD80, or CD86 after CAR T cell treatment equipped with CAR (1^st^ Gen), CAR (2^nd^ Gen), or CAR/CCR constructs (Figure 3E).

### CAR/CCR T cells show improved survival as a second-line treatment after conventional CAR T (2^nd^ Gen) therapy in xenograft lymphoma model

**Figure 4:**
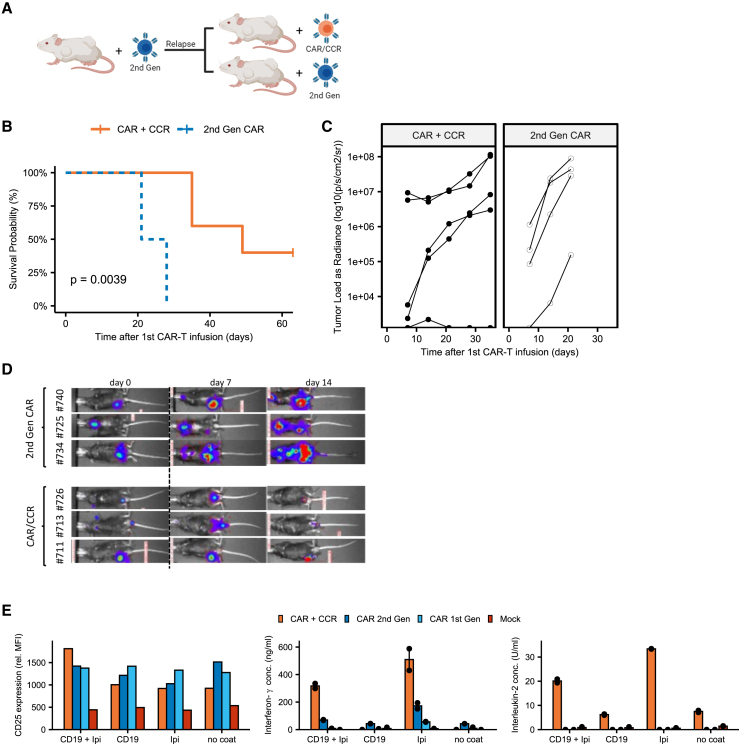
CAR/CCR T cells show improved survival as a second line treatment after conventional CAR T (2^nd^ Gen) therapy in xenograft lymphoma model (A) Graphical representation of second-line xenograft mouse trial protocol. Rag2^tm1.1Flv^ Il2rg^tm1.1Flv^ (Rag2^–^ γc^–^) received a Raji lymphoma intravenous xenograft (n = 22) and were treated with first-line (2^nd^ Gen) CAR T cells. Mice showing tumor relapse (n = 9) were divided into groups and treated with either CAR/CCR T cells (n = 5) or CAR (2^nd^ Gen) T cells (n = 4). (B) Kaplan-Meier plot of second-line mouse trial. Significance is evaluated via log-rank test and p value reported. (C) Luminescence plot describing tumor burden. (D) Representative selection of mice pictures with luminescence overlay describing tumor burden. (E) CD25 expression and cytokine secretion of stimulated CAR (+CCR) and untransduced T cells 24 days after first activation. Bar plots represent mean fluorescence intensity of pooled (n = 2) samples and mean of supernatant cytokines).

After demonstrating *in vivo* efficacy of our CAR/CCR construct in the treatment of CD19/CD80/CD86-positive tumors, we next raised the question whether second-line treatment with CAR (2^nd^ Gen) or CAR/CCR T cells can increase the survival of tumor-relapsed mice after CAR T cell treatment. For this purpose, Rag2^–^ γc^–^ mice (n = 22) were intravenously injected with CD19/CD80/CD86-positive Raji lymphoma cells and treated with CAR (2^nd^ Gen) T cells, corresponding to the Kymriah product (Novartis). While some of these mice experienced a long-lasting complete response, nine mice developed a rapidly growing tumor relapse. These were then divided into two groups to be treated either *de novo* with T cells expressing the CAR (2^nd^ Gen) or CAR/CCR construct (Figure 4A). CAR/CCR T cells significantly prolonged the survival of tumor relapsed mice compared with CAR (2^nd^ Gen) T cells (Figure 4B). While all tumor relapsed mice (4/4) treated with CD19 redirected T cells (2^nd^ Gen) died within 21 days after second-line treatment, 2/5 mice treated with CD19-specific CAR/CCR T cells were still alive at day 63 (Figures 4B–4D). Spleens of mice were frozen and sectioned and slides were stained via immunohistochemistry to visualize expression of CD19 (Figure S4D). Although organ availability and material quality were limited, human CD19 could be stained on the surface of lymphocytes, confirming that no primary target loss had taken place.

To understand why CAR/CCR T cells performed better than CAR (2^nd^ Gen) T cells, we analyzed cells that were kept in culture for up to 4 weeks (24 days after activation). We repeated (Figure 2F) the analysis of CD25 expression as a marker of 4-1BB activity, complemented with cytokine detection. After stimulation with recombinant CD19 and the anti-CTLA-4 antibody ipilimumab, CD8^+^ CAR/CCR T cells showed notably increased expression of CD25 compared with CAR (2^nd^ Gen) T cells, in contrast to experiments performed at 11 days after activation. CAR/CCR T cells also secreted much more IFN-γ and IL-2 than all other tested constructs, confirming that CAR/CCR T cells can be specifically activated and therefore be effective for longer periods of time.

We also adapted approaches to study the way co-receptors influence mitochondrial biogenesis reported by Kawalekar et al.^37^ and to study if the 4-1BB domain behaves differently in our constructs via western blot analysis of the phosphorylation of IKKα/β, previously reported by Gomes-Silva et al.^38^ Mitochondrial biogenesis analysis via qRT-PCR of stimulated and cultured cells revealed no significant differences between CAR/CCR and CAR (2^nd^ Gen) T cells after 7 and 14 days post-stimulation (Figure S4A). The phosphorylation of IKKα/β was high even in mock cells even after 2 weeks of post-transduction low-cytokine culture, most likely due to the intense stimulation with OKT3 and IL-2 that our transduction demands. This made it impossible to assess differences in signaling specific to CCR-ligand interaction (Figure S4C).

### CAR/CCR T cells reveal lower IL-2 and IL-6 secretion, and reduced activation when co-cultured with healthy B cells or CD19^+^ MSC-derived cells

**Figure 5:**
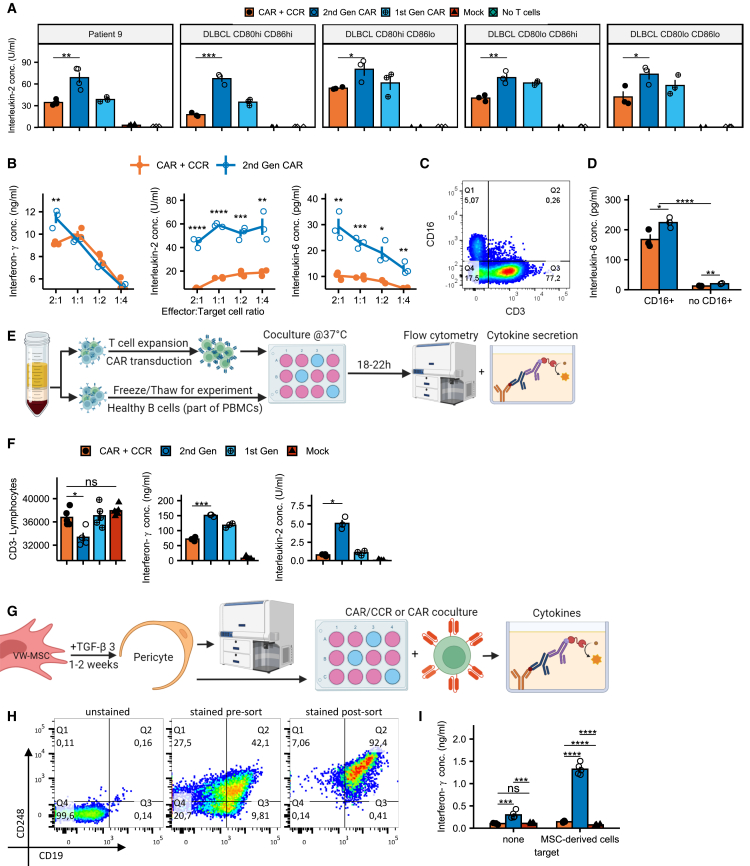
CAR/CCR T cells reveal reduced IL-2 and IL-6 secretion when co-cultured with tumor cells and reduced activation when co-cultured with healthy B cells or CD19^+^ MSC-derived cells (A) IL-2 secretion of CAR/CCR and control CAR T cells in co-culture with tumor cell lines and patient tumor cells. Bar plots represent mean ± SEM, n = 3/4. (B) Cytokine secretion across effector-target cell number ratios. Line plots represent mean ± SEM, n = 3; unmarked comparisons are not significant. (C) Flow cytometry plot of PMBCs expanded according to the transduction protocol stained to show CD16^+^ cells capable of secreting IL-6. See Figure S3E for gating strategy. (D) IL-6 secretion of PBMCs comparing samples containing CD16^+^ with samples without added CD16^+^ cells. Bar plots represent mean ± SEM, n = 3. (E) Graphical representation of healthy B cell depletion and cytokine secretion assay co-culturing transduced CAR T cells with heterogeneous PBMC samples from the same buffy coat containing healthy B cells. (F) Depletion of healthy B cells and associated cytokine secretion in co-culture. Bar plots represent mean ± SEM, n = 4/5 for flow cytometry, n = 3 for ELISA). (G) Graphical representation of VW-MSC differentiation and co-culture assay procedure. (H) FACS plots of post-differentiation VW-MSC-derived pericytes before and after MACS-based separation. Virtually all CD19^+^ cells also express the pericyte marker CD248 (see also Figure S4B). (I) IFN-γ secretion as a marker for CD19-directed CAR-induced activation comparing CAR/CCR, CAR (2^nd^ Gen), and mock T cells. Bar plots represent mean ± SEM, n = 4). Statistical significance was evaluated using a two-sided unpaired non-adjusted t test and significance levels reported according to p values: ∗p ≤ 0.05; ∗∗p ≤ 0.01; ∗∗∗p ≤ 0.001; ∗∗∗∗p ≤ 0.0001.

Expanding on the analysis of performance against lymphoma cell lines (Figures 2G and 2I), we studied the secretion of IL-2 and IL-6 by CAR/CCR and CAR T cells. While T cells equipped with CAR/CCR or CAR (2^nd^ Gen) were equally efficiently activated to release IFN-γ, the IL-2 release by activated CAR/CCR-redirected T cells was significantly reduced compared with T cells grafted with CAR (2^nd^ Gen) constructs (Figure 5A). As demonstrated in Figure 5B, these initial results remain constant for CAR/CCR and CAR (2^nd^ Gen) constructs when monitoring the release of IFN-γ and IL-2 across different effector to target ratios.

Surprisingly, increased IL-6 amounts were also detected in the co-culture supernatants of CAR (2^nd^ Gen) T cells co-cultivated with cells of the DLBCL line SU-DHL-10 (Figure 5B). To explain this result, we analyzed the CAR/CCR T cells for the presence of monocytes/macrophages by flow cytometry. As shown in Figures 5C and S3E, CAR- and CAR/CCR-redirected PBLs used in co-culture experiments were contaminated with ∼5% CD16^+^ monocytes, which upon activation represent a potential source of IL-6. We then performed co-culture experiments using CAR(/CCR) T cells, the B cell lymphoma cell line SU-DHL-10 and macrophages in a 3:1:1 ratio (CAR/lymphoma/monocytes). IL-6 secretion was indeed dependent on the presence of CD16^+^ monocytes (Figure 5D). Confirming the findings in Figure 5B, monocytes co-cultivated with CD19^+^CD80^high^CD86^high^ tumor cells and CAR (2^nd^ Gen) T cells were activated to secrete significantly more IL-6 than monocytes that were co-cultivated with CAR/CCR T cells and tumor cells.

Next, we investigated the effect of CAR/CCR-redirected T cells on non-tumor B cells, which usually express CD19 but no CD80 or CD86 on their surface when not activated (Figure 1C). CAR/CCR, CAR (2^nd^ Gen), and CAR (1^st^ Gen) T cells were co-cultivated with donor-matched primary B cells (Figure 5E). Elimination of non-tumor B cells was analyzed by performing a flow cytometry-based cytotoxicity assay and T cell-mediated release of the pro-inflammatory cytokines IL-2 and IFN-γ was determined using ELISA. As demonstrated in Figure 5F, CAR (2^nd^ Gen) T cells significantly reduce the CD19^+^ B cell population compared with T cells equipped with CAR/CCR or CAR (1^st^ Gen). In contrast to the CAR (2^nd^ Gen) T cells, the proposed CAR/CCR T cell strategy might therefore prevent an adverse effect on the viability of non-malignant B cells. The similar performance of CAR (1^st^ Gen) T cells lacking a 4-1BB co-stimulatory domain reflects that this is due to the lack of a co-stimulating 4-1BB signal in CAR/CCR T cells in the absence of CD80/86. This is also underpinned by the cytokine data presented in Figure 5F. CAR (2^nd^ Gen) T cells secreted significantly more IFN-γ and IL-2 than T cells with CAR/CCR or CAR (1^st^ Gen) receptors, which both produced IL-2 at very low levels.

As was recently reported by Parker et al.^11^ that a small population of CD19-positive cells might be implicated in CD19 CAR T cell-mediated neurotoxicity, we included this cell population in our selectivity study. Primary human vascular wall-typical mesenchymal stem cells (VW-MSCs)^39^ were differentiated into CD19^+^CD248^+^ pericytes by culture in medium supplemented with TGF-β3 for 2–6 weeks (Figures 5G and S4B). CD19^+^CD248^+^ cells were isolated to obtain a pure pericyte population to confirm the phenotype (Figure 5H). Finally, CAR (2^nd^ Gen) and CAR/CCR T cells were co-cultivated with CD19^+^CD248^+^ pericytes for 24 h. As demonstrated in Figure 5I, only CAR (2^nd^ Gen) T cells, but not CAR/CCR T cells, were activated for IFN-γ secretion by CD19 pericytes.

### CAR/CCR T cell model decreases CD80/86 positivity rate in an autochthonous lymphoma mouse model

**Figure 6:**
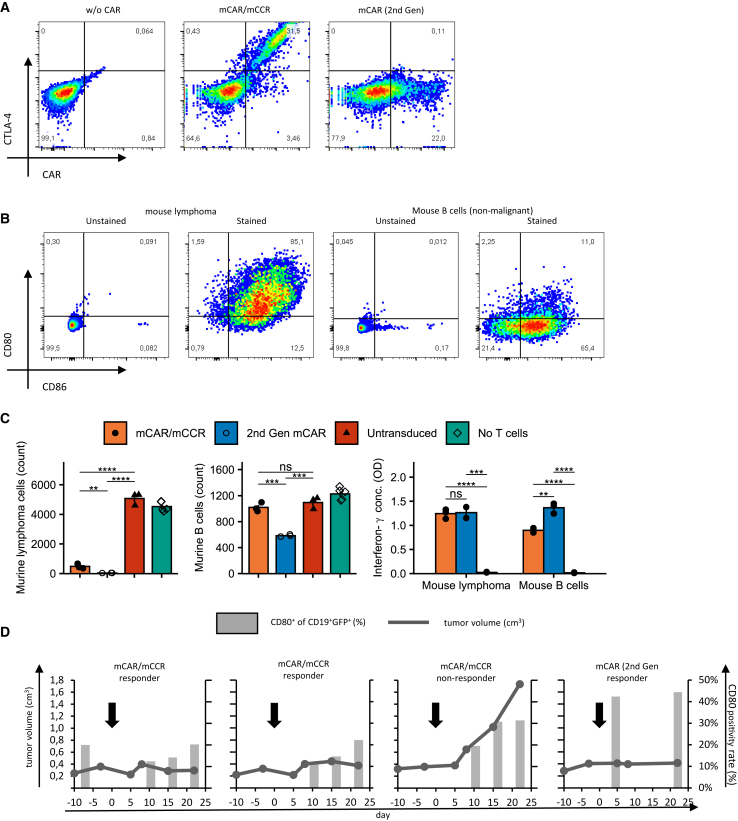
CAR/CCR T cell model decreases CD80/86 positivity rate in an autochthonous lymphoma mouse model (A) Representative FACS plots showing expression of the mCAR and mCAR/mCCR. (B) Representative FACS plots showing expression of CD19, CD80, and CD86 on a stable mouse lymphoma cell line and non-malignant murine B cells used in this study. (C) Results of *in vitro* co-culture assays with mCAR (2^nd^ Gen) and mCAR/mCCR with the lymphoma cell line and healthy B cells. Bar plots represent mean ± SEM, n = 3. (D) Results of *in vivo* selectivity trial showing tumor size and CD80 positivity rate of B cells over time. For gating strategy see Figure S5C. Statistical significance was evaluated using a two-sided unpaired non-adjusted t test and significance levels reported according to p values: ∗p ≤ 0.05; ∗∗p ≤ 0.01; ∗∗∗p ≤ 0.001; ∗∗∗∗p ≤ 0.0001.

To validate the selectivity of the CAR/CCR T cells, we translated our constructs into murine (m) mCAR (2^nd^ Gen) and mCAR/mCCR constructs, with extracellular domains specific to murine CD19, CD80, and CD86 and an intracellular murine CD3ζ domain. Due to the ineffectiveness of the 4-1BB intracellular domain in mice, we replaced 4-1BB with the murine CD28 co-stimulatory intracellular domain, retaining the basic strategy of the CAR/CCR switch. Murine CD3^+^ T cells were genetically modified to express fully murine aCD19-CD28^−^CD3ζ CAR and aCD19-CD3/CTLA-4-CD28 CAR/CCR constructs. Both constructs were efficiently expressed on the T cell surface (Figure 6A) and showed specific and notable activation and cytolytic activity (Figure 6C) when targeting a stable lymphoma cell line (CD19^+^, CD80^+^, CD86^+^) derived from Prdm1.fl/Myd88/Bcl2 mice^40^ (Figure 6B). As already observed in a human model, *in vitro* activity against non-malignant B cells isolated from mouse spleens was significantly reduced in the mCAR/mCCR T cells compared with mCAR (2^nd^ Gen) cells (Figure 6C). Interferon-γ secretion by the mCAR/mCCR T cells was also significantly decreased compared with mCAR (2^nd^ Gen), but still higher than in untransduced cells, suggesting some specific activation.

When injected into the Prdm1.fl/Myd88/Bcl2 mice after they had developed their autochthonous B cell lymphomas, mCAR and mCAR/mCCR cells prevented tumor growth in most mice. The proportion of CD19^+^/CD80^+^ B cells in the peripheral blood of these mice was notably lower in mCAR/mCCR-treated mice compared with the mouse treated with mCAR (2^nd^ Gen), meaning that more CD19^+^/CD80^–^ cells survived the treatment (Figures 6D and S5D). We also injected the murine constructs into immunocompetent C57BL/6 mice without lymphomas, observing almost absolute depletion of non-malignant B cells in the peripheral blood and spleen by mCAR (2^nd^ Gen), but not mCAR/mCCR T cells (Figure S5A). Interestingly, the few B cells spared by mCAR (2^nd^ Gen) T cells were predominantly expressing CD80/86 (Figure S5B), possibly indicating an escape from mCAR-mediated killing via CTLA-4-ligand interaction. We could also observe notable engraftment of mCAR (2^nd^ Gen) but not mCAR/mCCR T cells (Figure S5C), verifying that the depletion was indeed due to mCAR-mediated cytotoxicity. The results of both experiments confirm that the selective effect of the (m)CAR/(m)CCR concept applies to an immunocompetent mouse model and that normal B cells are spared in wild-type mice.

## Discussion

It is still a major challenge for CAR engineers to increase the safety and efficacy of CAR T cells so that they predominantly eliminate malignant cells with high selectivity and spare normal cells. The expression of most tumor-associated antigens is not limited to tumor cells leading to the risk of on-target/off-tumor effects. This is especially true for CD19 as it is not only expressed on leukemic cells but also on healthy B cells or brain mural cells.^11^ Consequently, anti-CD19 CAR T cell therapy does not only lead to the elimination of leukemic cells but also of healthy B cells and perivascular brain mural cells (pericytes), potentially causing B cell aplasia^41^ and neurotoxicity.^11^ To overcome this deficit, we designed the CAR/CCR concept to discriminate between healthy and tumor cells in B cell malignancies. We were supported by our discovery that malignant cells from DLBCL patients frequently express one or even both immune checkpoint ligands CD80 and CD86 before and after CAR T cell therapy. We therefore engineered two recombinant constructs, one (CAR) that recognizes CD19 and a second (CCR) with an extracellular CTLA-4 domain binding to CD80/86. The presented CD19-specific CAR construct corresponds to a 1^st^ Gen CAR that lacks a co-stimulatory domain for full T cell activation. The necessary co-activating signaling unit (4-1BB) is located in the co-expressed CTLA-4 CCR construct. By splitting the activating and co-stimulatory unit, we generated an “AND” switch mechanism that results in full activation of CAR/CCR T cells only when they bind to both target antigens. By allowing the transgenic CTLA-4 CCR construct to simultaneously compete for binding to its CD80/86 ligands with endogenous CTLA-4, we also aimed to increase the efficacy of CD19-CAR-T cell therapy. Surprisingly, to date there are no studies on CD80/86 in NHLs such as DLBCL and their impact on (CAR) T cell activity. The central role of CD80 and CD86 in the control of T cell activity has already been studied in detail. As early as 1996, Allison and colleagues demonstrated that antibodies directed against a cell surface molecule on T cells, CTLA-4, could trigger an immune response that cured mice of tumors.^42^

Another example of a switch concept in the field of B cell lymphoma was demonstrated by Blaeschke et al.^43^ The authors characterized a fully human PD-1-CD28 fusion protein in combination with anti-CD19 and anti-CD22 CAR constructs. However, in contrast to stable CD80 and/or CD86 expression on primary DLBCL cells, PD-L1 cell surface expression on pediatric B-cell precursor acute lymphoblastic leukemia (ALL) depends on induction by IFN-γ and TNF-α. The authors demonstrated that induction of primary blasts with these Th1 cytokines showed an interindividual heterogeneous response with upregulation, downregulation, or even no PD-L1 expression. Therefore, PD-L1 would be a poor target for the proposed CAR/CCR concept if a portion of tumor cells were negative for its expression and could escape elimination. Mansour et al. demonstrated that ALL patients who have received standard therapy can suffer a relapse associated with high expression of the CTLA-4 ligand CD86.^44^ Patients who died from the disease (9 patients) showed significantly higher CD86 expression and soluble CTLA-4 levels than surviving patients (51 patients).^44^ Since CTLA-4-mediated inhibition is crucial for T cell activity, immunomodulation via blockade of this pathway is a promising approach to prevent inactivation of tumor-reactive T cells. However, the clinical benefit of immunotherapy based on CTLA-4-specific antibodies in B cell lymphoma diseases, even in combination with other monoclonal antibodies, is low.^45^ In addition, CTLA-4/CD80/CD86-blocking antibodies can trigger autoimmune side effects through uncontrolled T cell proliferation of auto-reactive T cells. In a phase III trial comparing the efficacy of a 10 mg/kg dose of ipilimumab with that of a 3 mg/kg dose administered on the same schedule in patients with previously treated advanced-stage melanoma, patients in the high-dose anti-CTLA-4 antibody (ipilimumab) group had an increased prevalence of grade ≥3 adverse events.^46^

In our *in vitro* experiments, we show that co-culture of T cells equipped with the CAR/CCR construct with CD19^+^ tumor cell lines with different CD80/86 expression patterns results in significantly lower IL-2 release after antigen-specific activation compared with T cells equipped with a 2^nd^ Gen CAR construct (Tisagenlecleucel). The secretion of IL-2 is typically considered essential for CAR T efficacy, but IL-2 plays a dual role in T cell homeostasis, on the one hand activating non-regulatory T cells and on the other maintaining the presence of anti-inflammatory T-regulatory cells.^47^^,^^48^^,^^49^ CAR/CCR T cell-mediated release of IL-6, a major mediator of life-threatening CRS and neurotoxicity, is also reduced compared with CAR (2^nd^ Gen), potentially decreasing the severity of these toxicities and the need for therapeutic intervention. Crucially, the decrease in secretion of these cytokines was not associated with a decreased IFN-γ release and on-tumor cytolytic effect. Rather, CAR/CCR T cells retained the ability to be specifically activated for longer periods. Taken together, these properties of CAR/CCR T cells may have a positive impact on the efficacy and safety of the proposed immunotherapeutic CAR/CCR strategy in patients.

Recently, Xue et al. reported that CAR T cells secreting anti-IL-6 scFv and IL1RA could self-neutralize an IL-6 storm. They maintain low levels of IL1B during CAR T cell therapy to minimize IL-6- and IL-1-associated cytokine toxicity and neurotoxicity without impairing therapeutic efficacy in patients with hematological malignancy.^50^ One of the problems may be that the authors chose to constitutively express the IL-1B and IL-6 blockers, which may permanently exert the neutralizing influence on these key pro-inflammatory cytokines, weakening the patient’s immunity to pathogens. Therefore, the proposed CAR/CCR strategy provides a more elegant solution to keep IL-6 release low during CAR T cell therapy.

Like tisagenlecleucel, we use 4-1BB as the co-stimulatory and CD3ζ as the main activation domain in the CD19 CAR/CTLA-4 CCR constructs for T cell activation. Long et al. revealed that 4-1BB co-stimulation ameliorates T cell exhaustion induced by tonic signaling of chimeric antigen receptors.^51^ This observation was also confirmed by Singh et al., who showed that a CD22-specific CAR construct with the 4-1BB autonomic signaling unit enhanced immune synapse formation, activation of pro-inflammatory genes, and superior effector function.^52^ On the other hand, in 4-1BB-deficient mice, Lee et al. observed that 4-1BB-deficient CD8 T cells displayed hyperresponsiveness, expanding more than wild-type cells and showed enhanced maturation attributes compared with wild-type cells.^53^ Our studies of 4-1BB activity revealed that the expression of CD25 as an activity marker depends on the co-involvement of the CAR and CCR constructs only in Switch CAR/CCR T cells and that 2^nd^ Gen CAR T cells exhibit tonic upregulation at day 11 independent of the target antigen. This upregulated CD25 expression returns to baseline levels when 2^nd^ Gen CAR T cells are cultured for up to 4 weeks. Restimulation with recombinant CD19 at that time did not increase CD25 expression, suggesting a loss of CAR (2^nd^ Gen) T cell response in long-term culture. On the other hand, CAR/CCR T cells show CAR/CCR-mediated upregulation of CD25 expression even after nearly 4 weeks in culture, suggesting long-term activity of Switch CAR/CCR T cells. We also observed tonic activation directly downstream of 4-1BB, with similar IKK-alpha phosphorylation levels to stimulated CAR T cells even in mock-transduced T cells, likely due to the initial stimulation necessary for transduction.

In our direct and second-line *in vivo* studies, we demonstrate the superior efficacy of the CAR/CCR concept in the treatment of CTLA-4 ligand-positive B cell malignancies. This addresses a relevant clinical need, since most patients relapse or progress after CAR T cell therapy and retreatment with 2^nd^ Gen CAR T cells is rarely successful, with only 19% of NHL patients showing complete responses in a study by Gauthier et al.^54^ The prolonged ability to be specifically activated, the more specific 4-1BB signaling, and the distinct cytokine profile provide compelling reasons for improvements in efficacy. Competition with endogenous CTLA-4 for the binding of CD80 and CD86 is also likely to be a factor. As demonstrated by Agarwal et al., a CTLA-4 knockout in 2^nd^ Gen aCD19 CAR T cells leads to enhanced anti-tumor activity *in vivo*. This approach is mechanistically interesting, but the permanent deactivation of a checkpoint brake holds the risk of adverse events, as exemplified by an increased cytokine secretion reported in the study.^55^

We also demonstrate the universality of the CAR/CCR concept with different binding domains, possibly enabling CAR/CCR after CAR (2^nd^ Gen) treatment even in the minority of patients with decreased post-CAR CD19 expression.^54^^,^^56^

The CAR/CCR strategy additionally protects against CD19-directed on-target off-tumor side effects both *in vitro* and *in vivo*, since binding to CD19 antigen alone is not sufficient to trigger full CAR/CCR-mediated T cell activation. Clinical application of the CAR/CCR T cells could therefore reduce the incidence and severity of B cell aplasia and ICANS with subsequent infections, needed for immunoglobulin substitution and prolonged in-patient stays. Improved selectivity could also allow new CAR/CCR constructs to be designed with increased reactivity against the primary target antigen, protecting against escape by antigen downregulation.^57^^,^^58^

In addition, the application of switch CAR/CCR T cell strategy to other areas such as B cell-mediated autoimmune diseases is also conceivable, as autoreactive B cells clusters with the most recently activated class-switched mBC (memory B cell) phenotype exhibit high CD80 and CD86 expression.^59^^,^^60^

In conclusion, the CAR/CCR concept constitutes a promising approach to CAR T cell treatment of B cell malignancies with the potential for future scientific studies to investigate its mechanistic intricacies and evaluate its real-world clinical benefits in translational trials.

### Limitations of the study

The main limitation of our study lies in the use of *in vitro* experiments and xenograft mouse models, as these cannot fully simulate the complex interactions of cells in a patient’s body. Patient-derived xenograft models could provide further insight into these interactions. Our study could also only compare CD80 and CD86 expression before and after CAR T cell treatment in a small number of patients. Transcriptomic analysis of larger cohorts would strengthen these data and demonstrate a possible relationship between CAR T treatment and checkpoint ligand overexpression. Future investigations should also expand on the molecular impact of separating stimulatory domains.

## STAR★Methods

### Key resources table

**Table:** 

REAGENT or RESOURCE	SOURCE	IDENTIFIER
**Antibodies**		

CD19 CAR FMC63 Idiotype Antibody, REAfinity™, Biotin	Miltenyi Biotec	Cat#130-127-349; RRID:AB_2923109
Biotin Antibody, PE, REAfinity™	Miltenyi Biotec	Cat#130-110-951; RRID:AB_2661378
Brilliant Violet 421(TM) anti-human CD152 (CTLA-4)	BioLegend	Cat#369605; RRID:AB_2616790
CD3 Antibody, anti-human, APC	Miltenyi Biotec	Cat#130-113-125; RRID:AB_2725953
Anti-CD19 APC (LT19)	ImmunoTools	Cat#21270196; RRID:AB_2923108
Rabbit Anti-TEM1/CD248 Polyclonal Antibody, FITC Conjugated	Bioss	Cat#bs-2101R-FITC; RRID:AB_11083094
Anti-human CD152 (CTLA-4) PE/Cyanine7	BioLegend	Cat#369614; RRID:AB_2632876
Anti-human CD3 BV421	BioLegend	Cat#317344; RRID:AB_2565849
Goat Anti-mouse IgG Fab-Biotin	SouthernBiotech	Cat#1015-08; RRID:AB_2794195
Horse Anti-mouse HRP	Cell Signaling Technologies	Cat#7076S; RRID:AB_330924
Anti-CD20 APC (LT20)	ImmunoTools	Cat#21279206; RRID:AB_2923110
CD80 (B7-1) Monoclonal Antibody (2D10.4), Biotin, eBioscience	Thermo Fisher Scientific	Cat#13-0809-82; RRID:AB_466513
B7-2/CD86 Antibody (SPM600)	Novus Biologicals	Cat#NBP2-44515; RRID:AB_2923113
CD3 Monoclonal Antibody (UCHT1), Alexa Fluor™ 532, eBioscience	Thermo Fisher Scientific	Cat#58-0038-42; RRID:AB_11218675
Alexa Fluor® 488 Streptavidin	BioLegend	Cat#405235
Alexa Fluor® 647 anti-human CD19	BioLegend	Cat#302220; RRID:AB_389335
B7-2/CD86 Antibody (BU63)	Novus Biologicals	Cat#NBP2-25208; RRID:AB_2923115
Mouse Anti-IFN-gamma Monoclonal Antibody, Unconjugated, Clone NIB42	BD Biosciences	Cat#551221; RRID:AB_394099
Mouse Anti-IL-2 Monoclonal Antibody, Unconjugated, Clone 5344.111	BD Biosciences	Cat#555051; RRID:AB_395672
Mouse Anti-IFN-gamma Monoclonal Antibody, Biotin Conjugated, Clone 4S.B3	BD Biosciences	Cat#554550; RRID:AB_395472
Mouse Anti-IL-2 Monoclonal Antibody, Biotin Conjugated, Clone B33-2	BD Biosciences	Cat#555040; RRID:AB_395666
Anti-APC MicroBeads	Miltenyi Biotec	Cat#130-090-855; RRID:AB_244367
CD3 Antibody, anti-human, PE, REAfinity™	Miltenyi Biotec	Cat#130-113-139; RRID:AB_2725967
Brilliant Violet 510™ anti-human CD5 Antibody	BioLegend	Cat#364018; RRID:AB_2565728
CD19 Antibody, anti-human, FITC, REAfinity™	Miltenyi Biotec	Cat#130-113-645; RRID:AB_2726198
APC/Fire™ 750 anti-human CD20 Antibody	BioLegend	Cat#302357; RRID:AB_2572125
Brilliant Violet 421™ anti-human CD80 Antibody	BioLegend	Cat#305222; RRID:AB_2564407
anti-human CD86 APC-conjugated	ImmunoTools	Cat#21480866; RRID:AB_2923116
anti-human CD80 PE-conjugated	ImmunoTools	Cat#21270804; RRID:AB_2923118
B7-2/CD86 Antibody (BU63) [Alexa Fluor® 532]	Novus Biologicals	Cat# NBP2-34569AF532; RRID:AB_2923133
PE anti-mouse CD80 Antibody, Clone16-10A1	BioLegend	Cat#104707; RRID:AB_313128
PE/Cyanine7 anti-mouse CD86, Clone GL-1	BioLegend	Cat#105014; RRID:AB_439783
APC anti-mouse CD152 Antibody, Clone UC10-4B9	BioLegend	Cat#106309; RRID:AB_2230158
Ultra-LEAF Purified anti-mouse CD28 Antibody, Clone 37.51	BioLegend	Cat#102116; RRID:AB_11147170
Ultra-LEAF(TM) Purified anti-mouse CD3epsilon antibody, Clone145-2C11	BioLegend	Cat#100340; RRID:AB_11149115
Brilliant Violet 421(TM) anti-mouse CD3epsilon antibody, Clone1452C11	BioLegend	Cat#100341; RRID:AB_2562556
Anti-mouse CD19 APC, Clone 1D3	Immunotools	Cat#22270196; RRID:AB_2938950
Biotin-SP-AffiniPure F(ab')2 Fragment Goat Anti-Rat IgG, F(ab')2 Fragment Specific (min X Hu,Bov,Hrs Sr Prot) antibody	Jackson ImmunoResearch Labs	Cat#112-066-072; RRID:AB_2338185
Brilliant Violet 510(TM) anti-mouse CD19 antibody, Clone 6D5	BioLegend	Cat#115545; RRID:AB_2562136
IFN gamma Monoclonal Antibody (AN-18), eBioscience, Clone AN-18	Thermo Fisher Scientific	Cat#14-7313-81; RRID:AB_468471
Biotin anti-Mouse IFN gamma	BD Pharmingen	Cat#551506; RRID:AB_394224
anti-*p*-IKK alpha/beta (S176 + S180)	Bioss	Cat#bs-3237R; RRID:AB_10883648
anti-Rabbit IgG HRP	Santa Cruz Biotechnology	Cat#sc-2030; RRID:AB_631747
Goat F(ab')2 Anti-Human IgG, Mouse ads-PE	Southern Biotech	Cat#2043-09; RRID:AB_2795669
Purified anti-human CD19 Antibody	BioLegend	Cat#302202; RRID:AB_314232
Biotin anti-mouse IgG1 Antibody	BioLegend	Cat#406603; RRID:AB_315062
G4S Linker (E7O2V) Rabbit mAb (Alexa Fluor ® 488 Conjugate)	Cell Signaling Technologies	Cat#50515S; RRID:AB_2941670
PE anti-human CD25	BioLegend	Cat#302606; RRID:AB_314276
CD3 Antibody, anti-human, FITC, REAfinity™	Miltenyi Biotec	Cat#130-113-138; RRID:AB_2725966
Brilliant Violet 510™ anti-human CD4	BioLegend	Cat#317444; RRID:AB_2561866
CD8 Antibody, anti-human, APC-Vio® 770, REAfinity™	Miltenyi Biotec	Cat#130-110-819; RRID:AB_2659246
Brilliant Violet 421™ anti-human CD3	BioLegend	Cat#317344; RRID:AB_2565849
APC anti-human CD27	BioLegend	Cat#356410; RRID:AB_2561957
PE/Fire™ 700 anti-human CD45RA	BioLegend	Cat#304171; RRID:AB_2888784)
FITC anti-human CD45RO	BioLegend	Cat#304204; RRID:AB_314420
APC/Fire™ 750 anti-human CD62L	BioLegend	Cat#304845; RRID:AB_2629675
PE anti-human CD4	BioLegend	Cat#317410; RRID:AB_571955
FITC anti-human CD8a	BioLegend	Cat#300906; RRID:AB_314110
PE/Cyanine7 anti-human TIGIT (VSTM3)	BioLegend	Cat#372713; RRID:AB_2632928
APC anti-human CD279 (PD-1)	BioLegend	Cat#329908; RRID:AB_940475
APC/Fire(TM) 750 anti-human CD366 (Tim-3)	BioLegend	Cat#345043; RRID:AB_2632855
Goat F(ab')2 Anti-Human IgG, Mouse ads-FITC	SouthernBiotech	Cat#2043-02; RRID:AB_2795666

**Chemicals, peptides, and recombinant proteins**		

Recombinant Human Transforming Growth Factor β 3	ImmunoTools	Cat#11344485
PEIpro®	Polyplus	Cat#101000026
Recombinant Human B7-2 Fc	Peprotech	Cat#310-33
Propidium Iodide Solution	Miltenyi Biotec	Cat#130-093-233
CellTrace Violet Cell Proliferation Kit	Invitrogen	Cat#C34557
Streptavidin-PE, SAv-PE	BioLegend	Cat#405203
7-AAD Viability Staining Solution	BioLegend	Cat#420404
Streptavidin-POD Conjugate	Roche Diagnostics	Cat#11089153001
Streptavidin FITC-conjugated	ImmunoTools	Cat#31274243
Recombinant Human CD19-Fc Chimera	BioLegend	Cat#789006
Human B7-1/CD80 Protein, Fc Tag (MALS verified)	Acro Biosystems	Cat#B71-H5259
Human B7-2/CD86 Protein, Fc Tag, premium grade	Acro Biosystems	Cat#CD6-H5257

**Critical commercial assays**		

ELISA MAX™ Deluxe Set Human IL-6	BioLegend	Cat#430504
MojoSort Mouse CD3 T cell Isolation Kit	BioLegend	Cat#480031
MojoSort Mouse Pan B Cell Isolation Kit II	BioLegend	Cat#480087
CellTrace™ Cell Violet Proliferation Kit	Invitrogen	Cat#C34557

**Experimental models: Cell lines**		

Human: Raji	DSMZ	Cat#ACC 319; RRID:CVCL_0511
Human: SU-DHL-10	DSMZ	Cat#ACC 576; RRID:CVCL_1889
Human: DOHH-2	DSMZ	Cat#ACC 47; RRID:CVCL_1179
Human: Oci-Ly1	DSMZ	Cat#ACC 722; RRID:CVCL_1879
Human: Oci-Ly19	DSMZ	Cat#ACC 528; RRID:CVCL_1878
Human: HEK-293	DSMZ	Cat#ACC 305; RRID:CVCL_0045
Human: Vascular Wall-Typical Mesenchymal Stem Cells (Hita08/20P5)	Diana Klein^39^	N/A
Murine: Prdm1.fl/Myd88/Bcl2-derived lymphoma cell line	Ruth Flümann^40^	N/A

**Experimental models: Organisms/strains**		

Mouse: Rag2tm1.1Flv Il2rgtm1.1Flv (Rag2- γc-)	The Jackson Laboratory	Cat#014593; RRID:IMSR_JAX:014593
Mouse: Prdm1^fl/fl^; Myd88^cond.p.L252P/wt^;Rosa26^LSL.BCL2.IRES.GFP/wt^;Cd19^Cre/wt^ (Prdm1.fl/Myd88/Bcl2)	Ruth Flümann^40^	N/A
Mouse: C57BL/6J (Black 6)	The Jackson Laboratory	RRID:IMSR_JAX:000664

**Recombinant DNA**		

Plasmid: pBullet_aCD19_CD3zeta_P2A_CTLA4_4-1BB	This paper	N/A
Plasmid: pBullet_aCD19_CD3zeta	This paper	N/A
Plasmid: pBullet_aCD19_CD3zeta_4-1BB	This paper	N/A
Plasmid: pBullet_aCD20_CD3zeta_4-1BB	This paper	N/A
Plasmid: pBullet_aCD20_CD3zeta_P2A_CTLA-4_4-1BB	This paper	N/A
Plasmid: pBullet_aCD19_CD3zeta_P2A_aCD86_4-1BB	This paper	N/A
Plasmid: pQCXIP_eGFP	This paper	N/A
Plasmid: pQCXIP_fLuc_tdTomato	This paper	N/A

**Software and algorithms**		

R code	This paper	https://doi.org/10.5281/zenodo.10342282
RStudio	RStudio Team	https://www.rstudio.com/
R Core	R Core Team	https://www.r-project.org/
ggpubr		https://cran.r-project.org/web/packages/ggpubr/index.html
Survival		https://cran.r-project.org/web/packages/survival/index.html
Survminer		https://cran.r-project.org/web/packages/survminer/index.html
rstatix		https://cran.r-project.org/web/packages/rstatix/index.html

**Other**		

Mesenchymal Stem Cell Growth Medium 2	PromoCell	Cat#C-28009
Canto II Flow Cytometer	Becton Dickinson	https://www.bdbiosciences.com/en-eu/products/instruments/flow-cytometers/clinical-cell-analyzers/facscanto
Multiskan GO Microplate Spectrophotometer	Thermo Scientific	https://www.fishersci.de/shop/products/multiskan-go-microplate-spectrophotometer/p-4530546
Hidex Sense Microplate Reader	Hidex	https://www.hidex.de/hidex-sense/
IVIS 200 Spectrum *In Vivo* Imaging System	PerkinElmer	https://www.perkinelmer.com/de/product/ivis-instrument-spectrum-120v-andor-c-124262
FV 1000 confocal laser scanning microscope	Olympus	https://www.olympus-lifescience.com/de/technology/museum/micro/2004/
UC90 4K microscope	Olympus	https://www.olympus-lifescience.com/de/camera/color/uc90/
BX53 microscope	Olympus	https://www.olympus-lifescience.com/en/microscopes/upright/bx53f2/
Fusion Solo S	Vilber	https://www.vilber.com/fusion-solo-s/
MACSQuant X	Miltenyi Biotec	https://www.miltenyibiotec.com/US-en/products/macsquant-x.html

### Resource availability

#### Lead contact

Further information and requests for resources and reagents should be directed to and will be fulfilled by the lead contact, Lars Fabian Prinz (lars.prinz1@uk-koeln.de).

#### Materials availability

Plasmids generated in this study will be available on request through completion of a Material Transfer Agreement.

#### Data and code availability

• There was no data gathered in this study that consists of Cell Press standardized datatypes. All data reported in this paper will be shared by the lead contact upon request.
• All original R code used for data analysis and visualization has been deposited at Zenodo and is publicly available as of the date of publication under https://doi.org/10.5281/zenodo.10342282.
• Any additional information required to reanalyze the data reported in this paper is available from the lead contact upon request.

### Experimental models and study participant details

#### Cell lines

Human lymphoma cell lines Raji (male; DSMZ Cat#ACC 319, RRID: CVCL_0511), SU-DHL-10 (male; DSMZ Cat#ACC 576, RRID: CVCL_1889), DOHH-2 (male; DSMZ Cat#ACC 47, RRID: CVCL_1179), Oci-Ly1 (male; DSMZ Cat#ACC 722, RRID: CVCL_1879) and Oci-Ly19 (female; DSMZ Cat#ACC 528, RRID: CVCL1878) were cultured in RPMI 1640 medium with 10% (Raji, DOHH-2) or 20% (SU-DHL-10, Oci-Ly1, Oci-Ly19) fetal bovine serum (FBS), 10mM HEPES buffer and 100U mL^−1^/100 μg mL^−1^ penicillin/streptomycin at 37°C and 5% CO_2_ in a humidity-controlled environment. Cultures were split and media exchanged every 3–4 days. Cultures were checked for mycoplasma via PCR at regular intervals and before *in vivo* application.

VW-MSCs were cultured in PromoCell Mesenchymal Stem Cell Growth Medium 2 at 37°C and 5% CO_2_ in a humidity-controlled environment. Cells were further differentiated with TGF-beta 3 (10–20 ng mL^−1^) and split at 70–90% confluence. Passages 7 through 11 were used in analyses.

The murine lymphoma cell line derived from Prdm1.fl/Myd88/Bcl2 mice was cultured in DMEM supplemented with 10% FCS, Pen/Strep, HEPES buffer, 1% Sodium-pyruvate, 1% MEM NEAA and 0.1% β-Mercaptoethanol at 37°C and 10% CO_2_ in a humidity-controlled environment. Cultures were split and media exchanged every 3–4 days.

#### Primary cell cultures

Primary lymphoma B cells were isolated from peripheral blood via gradient isolation of PBMCs and cultured short-term in RPMI 1640 medium with 10% FBS, 10mM HEPES buffer and 100U mL^−1^/100 μg mL^−1^ penicillin/streptomycin at 37°C and 5% CO_2_ in a humidity-controlled environment. Co-cultures with (CAR or CAR/CCR) T cells were cultured under the same conditions.

Primary healthy peripheral blood mononuclear cells (PBMCs) were derived from blood donor buffy coats via gradient isolation and either used as is or subjected to a T cell expansion procedure in preparation for transduction. Collection and scientific use of donor buffy coats was consented by the donors and approved by the ethical review committee of the University of Cologne Medical Faculty under ref. 21–1317. PBMCs were cultured in RPMI 1640 media with 10% FCS, HEPES buffer, Pen/Strep and 1000 U mL-1 IL-2, 200 ng mL-1 OKT3 and 50 ng mL-1 15E8 at 37°C and 5% CO_2_ in a humidity-controlled environment.

Post-transduction T cells were cultured in RPMI 1640 media with 10% FCS, HEPES buffer, Pen/Strep and 100-300U mL^−1^ IL-2 at 37°C and 5% CO_2_ in a humidity-controlled environment. Culture media was added or exchanged every 3–4 days or when beginning acidification was observed via phenol red color change. Prior to assays stimulation was removed by media exchange the day before the assay.

Donor gender cannot be reported because it was not supplied with samples or buffy coats.

#### Mouse models

For the first-line efficacy xenograft mouse trial, a total of 25 treatment-naïve adult Rag2^tm1.1Flv^ Il2rg^tm1.1Flv^ (Rag2- γc-; RRID: IMSR_JAX:014593) mice between the ages of 100–147 days (12 female, 13 male) were intravenously injected with Raji-fLuc cells (5x10^4^ cells per mouse) on day −3. One female mouse was injected intraperitoneally. Tumor engraftment was evaluated on day 3 by injecting D-Luciferin (1.5 mg per mouse) and measuring tumor luminescence in anesthetized mice in a IVIS200 device (PerkinElmer, Waltham, Massachusetts, USA). Mice were randomly split into groups receiving 8x10^6^ cells each of the CAR/CCR (6 mice, 5 female), Second-generation CAR (8 mice, 4 female) or untransduced T cells (9 mice, 2 female) from the same PBMC donor. 2 mice (1 female) remained untreated, one of which was the mouse injected intraperitoneally. All mice were scored daily with luminescence measurements repeated weekly. Mice were sacrificed at predetermined scoring cutoff.

For the second-line efficacy xenograft mouse trial, a total of 22 treatment-naïve adult Rag2^tm1.1Flv^ Il2rg^tm1.1Flv^ (Rag2- γc-; RRID: IMSR_JAX:014593) mice between the ages of 88 and 179 days (9 female, 15 male) were intravenously injected with Raji-fLuc cells (5x10^5^ cells per mouse) on day −5. Tumor engraftment was evaluated on day −1 and all mice treated with CAR (2^nd^ Gen) T cells on day 0. 2 male mice died before reevaluation. 9 mice (1 female) between the ages of 106–179 days at tumor infusion experienced relapses between day 13 and 20 and were randomly assigned to be treated with 5x10^6^ mCAR/mCCR T cells (5 mice, all male) or mCAR (2^nd^ Gen) T cells (4 mice, 1 female) on day 19 or 26 depending on relapse date. All mice were scored daily with luminescence measurements repeated weekly. Mice were sacrificed at predetermined scoring cutoff.

For the mouse trial investigating the activity against CD80/86 negative cells in mice, a total of 4 Prdm1^fl/fl^; Myd88^cond.p.L252P/wt^;Rosa26^LSL.BCL2.IRES.GFP/wt^;Cd19^Cre/wt^ (Prdm1.fl/Myd88/Bcl2) mice were monitored with MRI scans for the development of a spontaneous lymphoma associated with their genotype and marked by splenomegaly.^40^ Once this was observed, 2x10^6^ CAR(/CCR) T cells were infused, and the mice monitored via peripheral blood samples and MRI for CD80/CD86 positivity rate of B cells and spleen size respectively.

Primary mouse B and T cells were isolated with Pan B or CD3 T cell isolation kits from singularized spleen tissue explanted from C57BL/6J (Black 6; RRID:IMSR_JAX:000664) mice and cultured in X-Vivo 15 medium at 37°C and 10% CO_2_ in a humidity-controlled environment. Post-transduction the X-Vivo 15 medium for mCAR and mCAR/mCCR T cells was supplemented with 500U mL^−1^ IL-2 (50U mL^−1^ on the day before tests) and 10 ng mL^−1^ IL-15.

For the investigation selectivity of mCAR(/mCCR) T cells in immunocompetent mice without lymphomas, two C57BL/6J (Black 6; RRID:IMSR_JAX:000664) mice were injected with either 2x10^6^ mCAR/mCCR or mCAR (2^nd^ Gen) T cells. Flow cytometric analysis of was performed on peripheral blood samples at baseline (−80 days) and 7, 15, 22 and 29 days after infusion, and on singularized spleen cells 30 days after infusion.

All animals were housed according to protocols approved by the Institutional Animal Use Committee of the State North Rhine-Westphalia (Germany) and maintained in pathogen-free conditions in a barrier facility.

All mouse trials were approved by the Landesamt für Natur, Umwelt und Verbraucherschutz Nordrhein-Westfalen under ref. 2020-A469 (Rag2- γc-), 2019.A457 (C57BL/6J) and 2022.A146 (Prdm1.fl/Myd88/Bcl2).

#### Patient derived samples

Detailed patient demographics and disease as well as treatment characteristics can be found in Figures S1B and S1C. Both surviving patients were male with a mean age of 57.5 years while the mean age of all patients was 60.5 years. Data collection was halted on 2021/12/28 for analysis and follow-up duration truncated at 400 days to improve plot legibility for patient 8, who at that point had a total follow-up duration of 761 days. Biopsy and clinical data analysis was consented by the patients and approved by the ethical review committee of the University of Cologne Medical Faculty under reference BioMaSota 13–091.

### Method details

#### CD80/86 transcript abundance analysis

Transcript abundances for CD80 and CD86 were queried from preprocessed and already normalized indicated published datasets (Brune et al.,^32^ Chapuy et al; ,^34^ Schmitz et al; ^33^).

#### Vector creation and preparation

Transfection vectors were ordered from Integrated DNA Technologies (IDT) as gBlocks, ligated and transformed into DH5-alpha Escherichia coli bacteria via heat shock. Transformed bacteria were cultured on selection agar containing Ampicillin, single colonies picked, expanded, and evaluated for correct plasmid configuration after plasmid preparation via restriction enzyme digestion and gel electrophoresis as well as Sanger DNA sequencing. Plasmids were then isolated as needed from transformed bacteria selectively cultivated in LB broth containing Ampicillin via NucleoBond Extra Midi Prep Kit performed according to the user manual.

#### PBMC isolation and T cell activation

Peripheral blood mononuclear cells were harvested from blood donor buffy coats via density gradient isolation. Buffy coats were carefully pipetted onto STEMCELL Technologies Lymphoprep and centrifuged at 600 x g for 30 min. The resulting mononuclear cell layer was extracted, washed 4 times in PBS and up to 1e9 cells put into culture in ThermoFisher RPMI 1640 medium with 10% FCS, Pen/Strep and HEPES buffer. T cells were stimulated with 1000 U mL^−1^ IL-2, 200 ng mL^−1^ OKT3 and 50 ng mL^−1^ 15E8 for two days before transduction.

Primary mouse cells were gathered by singularizing the spleen of C57BL/6J (Black 6; RRID:IMSR_JAX:000664) mice and isolating either B cells with a MojoSort Mouse Pan B Cell Isolation Kit II (BioLegend Cat#480087) or T cells with a MojoSort Mouse CD3 T cell Isolation Kit (BioLegend Cat#480031) according to the manufacturer’s instructions. Murine B cells were cultured in RPMI 1640 supplemented with 10% FCS and Pen/Strep, murine T cells were cultured in X-Vivo 15 supplemented with 5% FCS and stimulated with 200 ng mL^−1^ anti-CD3, 100 ng mL^−1^, 1000U mL^−1^ IL-2 and 10 ng mL^−1^ IL-15 for three days prior to transduction.

#### Vector transfection and transduction

CAR and CAR/CCR expression was induced by retroviral transduction. 10cm plates with a monolayer of HEK293t cells were transfected at 50–70% confluence using 20μL Polyplus PeiPro transfection reagent with 10μg construct vector DNA and 5μg each of pCOLT-GAL-V and pHIT60 (MoMuLV) plasmids in 500μL RPMI 1640. Transfection reagents were added onto the HEK293t cells cultured in 9mL of RPMI 1640 with 10% FCS, Pen/Strep and HEPES buffer. The culture medium containing transduction viruses was harvested after 10–24 h and added to 1.6e7 PBMC-derived T cells in a plate coated with poly-D-Lysin, centrifuged at 1600 x g for 90 min and cultured overnight. The process was repeated a second time with the same cells to improve transduction yields. During transduction T cells were kept stimulated overnight with 1000 U mL^−1^ IL-2 after the first, and 500–800 U mL^−1^ IL-2 after the second run.

mCAR and mCAR/mCCR expression was also induced by retroviral transduction similar to the process detailed for human PBMC. The X-Vivo 15 media used were supplemented with 5% FCS and 10 μg mL^−1^ protamine sulfate, and cells stimulated with 500U mL^−1^ IL-2 and 10 ng mL^−1^ IL-15. The virus containing medium was centrifuged onto 6-well plates coated with Poly-D-Lysin at 1200 x g for 90 min after which the cells were added.

#### CAR/CCR detection via flow cytometry

Transduction efficiency was evaluated via FACS analysis using anti-idiotype CD19-CAR (FMC63) (Miltenyi Biotec Cat#130-127-349; RRID:AB_2923109), anti-mouse Fab Biotin (Southern Biotech Cat#1015-08; RRID:AB_2794195) or anti-G4S Linker (Cell Signaling Technology Cat#71645S; RRID:AB_2941670) antibodies and detected with anti-Biotin PE (Miltenyi Cat#130-110-951; RRID:AB_2661378). CCRs were stained with anti-CTLA4 (CD152) BV421 (BioLegend Cat#369605; RRID:AB_2616790), anti-CTLA PE/Cy7 (BioLegend Cat#369614; RRID:AB_2632876) or with human B7-2 (CD86) Fc-linked recombinant protein (Peprotech Cat#310-33), marked with anti-hIgG PE (Southern Biotech Cat#2043-09; RRID:AB_2795669). CD3 was stained with anti-CD3 APC (Miltenyi Cat#130-113-125; RRID:AB_2725953. Antibodies were incubated for 30 min at 4°C with two PBS washing steps each between primary and secondary antibodies and before analysis. CAR Transduction efficiency was evaluated in a Lymphocyte/Single Cell/CD3+ gate to confirm successful transduction and normalize CAR T cell numbers in downstream experiments.

Antibodies used for the evaluation of mCAR(/mCCR) transduction efficiency were anti-mouse CD3epsilon (1452C11) BV421 (BioLegend Cat#100341; RRID:AB_2562556), anti-mouse CD152 (UC10-4B9) APC (BioLegend Cat#106309; RRID:AB_2230158) and Biotin-SP-AffiniPure F(ab’)2 Fragment Goat anti-Rat IgG (Jackson ImmunoResearch Labs Cat#112-066-072; RRID:AB_2338185) antibodies, the last of which was marked with Streptavidin-PE (BioLegend Cat#405203).

Reagents used for the evaluation of CD80/CD86 binding of CAR and CCR receptors on transfected HEK 293t cells were Fc-tagged Human B7-1/CD80 Protein (Acro Biosystems Cat#B71-H5259) or Human B7-2/CD86 Protein (Acro Biosystems Cat#CD6-H5257), detected with FITC-linked Goat F(ab')2 Anti-Human IgG (SouthernBiotech Cat#2043-02; RRID:AB_2795666). Analysis was performed on transfected HEK 293t cells to control for endogenous expression of CD28 and/or CTLA-4.

#### 4-1BB activation detection via CD25 expression

Transduced CAR and CAR/CCR T cells were cultured on microwell plates with plate-bound anti-mouse Fab antibody (Southern Biotech Cat#1015-08; RRID:AB_2794195) and/or Ipilimumab at 5μg/ml respectively. Anti-mouse Fab antibody was replaced by recombinant CD19-Fc chimeric protein (BioLegend Cat#789006) in one experiment studying cells 24 days after activation, to enable cytokine analysis in the supernatant. Cells were harvested after 24 h and stained for flow cytometry-based detection with anti-CD3 (REA613) FITC (Miltenyi Biotec Cat#130-113-138; RRID:AB_2725966), anti-CD4 (OKT4) BV510 (BioLegend Cat#317444; RRID:AB_2561866), anti-CD8 (REA734) APC-Vio770 (Miltenyi Biotec Cat#130-110-819; RRID:AB_2659246), anti-mouse Fab Biotin (Southern Biotech Cat#1015-08; RRID:AB_2794195) detected with Streptavidin-APC (BioLegend Cat#405243), and anti-CD25 (BC96) PE (BioLegend Cat#302606; RRID:AB_314276) antibodies. Mean fluorescence of CD25 expression in CD3^+^ CAR+ CD8+cells was determined on the Canto II (Becton Dickinson, Franklin Lakes, USA) platform.

#### Differentiation and checkpoint receptor expression

PBMC from healthy donors where isolated, activated and transduced as described. Analysis of differentiation status and expression of checkpoint receptors was performed on the day of transduction (“d4”) and after 5 days of cultivation in retroviral supernatants (“d9”). Cells were harvested and stained for flow cytometry-based detection of differentiation markers with anti-CD3 (OKT3) BV421 (BioLegend Cat#317344; RRID:AB_2565849), anti-mouse Fab Biotin (Southern Biotech Cat#1015-08; RRID:AB_2794195) detected with Streptavidin-PE (BioLegend Cat#405243), anti-CD27 (M-T271) APC (BioLegend Cat#356410; RRID:AB_2561957), anti-CD45RA (HI100) PE/Fire700 (BioLegend Cat#304171; RRID:AB_2888784), anti-CD45RO (UCHL1) FITC (BioLegend Cat#304204; RRID:AB_314420), and anti-CD62L (DREG-56) APC/Fire750 (BioLegend Cat#304845; RRID:AB_2629675) antibodies. For flow cytometry-based detection of checkpoint receptors, cells were stained with anti-CD4 (OKT4) PE (BioLegend Cat#317410; RRID:AB_571955), anti-CD8 (HIT8a) FITC (BioLegend Cat#300906; RRID:AB_314110), anti-TIGIT (A15153G) PE/Cy7 (BioLegend Cat#372713; RRID:AB_2632928), anti-CD279 (PD-1) (EH12.2H7) APC (BioLegend Cat#329908; RRID:AB_940475), and anti-CD366 (TIM-3) (F38-2E2) APC/Fire750 (BioLegend Cat#345043; RRID:AB_2632855) antibodies. Differentiation of CD3^+^ CAR+ cells and checkpoint receptor expression on CD4^+^ and CD8^+^ cells was determined on the Canto II (Becton Dickinson, Franklin Lakes, USA) platform.

#### GFP/tdTomato transduction of tumor cell lines

Stable eGFP expression was induced in tumor cell lines via retroviral transduction. Two 10cm plates with a monolayer of HEK293t cells were transfected at 50–70% confluence with 20μL Polyplus PeiPro transfection reagent with 10μg DNA of plasmid pQCXIP_eGFP and 5μg each of pHIT60 (MoMuLV) and pMD2.G (VSV-G envelope) in 500μL RPMI 1640. Culture supernatant containing transduction viruses was harvested after 10–24 h, added to 5x10^5^ tumor cells, centrifuged at 1600 x g for 90 min and cultured for several days. Plasmid expressing cells were selected via puromycin treatment and fluorescence confirmed via flow cytometry on the Canto II (Becton Dickinson, Franklin Lakes, USA) platform (SuppFigure 2D).

Stable tdTomato/fLuc expression was induced in Raji cells via retroviral transduction applying the same procedure, substituting the eGFP plasmid with 10μg of pQCXIP_fLuc_tdTomato.

#### Fluorescence based cytotox assays

Cytolytic activity of studied CAR and CAR/CCR T cells was evaluated by measuring fluorescence levels over time in co-cultures of T cells and eGFP/tdTomato-expressing Tumor cell lines using the HIDEX Sense (Hidex, Turku, Finland) microplate reader platform with the compatible digital atmospheric control. Cytolytic activity is given as inverse relative fluorescence level increase compared to measurements of only tumor containing wells, calculated using the formula:

$${CyTox}_{i}\left[\%\right]=\left(1-\frac{\frac{\left({Fl}_{i}-\;{Med}_{i}\right)}{\left({Fl}_{0}-{Med}_{0}\right)}}{\frac{\left({Tu}_{i}-\;{Med}_{i}\right)}{\left({Tu}_{0}-\;{Med}_{0}\right)}}\;\right)x100$$

Where Fl = Fluorescence measurement in co-culture wells, Tu = Fluorescence measurement in wells containing only tumor cells and Med = Fluorescence measurements in wells containing only the cultivation medium. Subscripted i and 0 signify the co-culture time in hours and the initial measurement respectively.

Assay wells contained 200μL RPMI 1640 with 10% FCS, Pen/Strep and HEPES, tumor cells and CAR T cells in different ratios, normalized to total CAR T cell numbers and total cell numbers by adding Mock-transduced T cells from the same donor.

#### Antibody-based *in vitro* cytotoxicity assays

Depletion of primary patient lymphoma cells was investigated after co-culture with CAR/CCR and CAR T cells with an effector-target ratio of 1:7 at 37°C in a humidity-controlled environment via flow cytometry using the Canto II platform (Becton Dickinson, Franklin Lakes, USA). Samples were stained with anti-CD3 PE (Miltenyi Biotec Cat#130-113-139; RRID:AB_2725967), anti-CD5 BV510 (BioLegend Cat#364018; RRID:AB_2565728), anti-CD19 FITC (Miltenyi Biotec Cat#130-113-645; RRID:AB_2726198), anti-CD20 APC-Fire750 (BioLegend Cat#302357; RRID:AB_2572125), anti-CD80 BV421 (BioLegend, Cat#305222; RRID:AB_2564407), anti-CD86 APC (ImmunoTools Cat#21480866; RRID:AB_2923116) and 7-AAD (BioLegend Cat#420404) antibodies, washed two times with PBS and then analyzed. Optimal assay duration was determined via preliminary analyses at different timepoints.

Depletion of non-tumor B cells was investigated after co-culture with CAR/CCR and CAR T cells for 18 h with an effector-target ratio of 1:10 at 37°C in a humidity-controlled environment via flow cytometry. Samples were stained with anti-CD3 APC (Miltenyi Biotec Cat#130-113-125; RRID:AB_2725953), anti-CD19 FITC (Miltenyi Biotec Cat#130-113-645; RRID:AB_2726198) and 7-AAD (BioLegend Cat#420404), washed twice and analyzed.

*In vitro* depletion of lymphoma cell line and primary murine B cells by mCAR/mCCR and mCAR (2^nd^ Gen) as well as SU-DHL-10 cells in experiments pictured in Figure S4 was detected by pre-staining the target cells with CellTrace Violet Proliferation Kit before adding the effector cells for 24–48 h. Cells were then stained with propidium iodide, detected using the MACSQuant X flow cytometer (Miltenyi Biotec, Bergisch-Gladbach, Germany) and absolute counts of cells alive CellTrace Violet positive cells plotted for comparison between constructs.

#### Cytokine detection via ELISA

Concentration of secreted Interferon-γ and Interleukin-2 in assay supernatants was measured using Sandwich-ELISA technology. Nunc MaxiSorp 96-well plates were coated with anti-human IFN-γ antibody (BD Biosciences Cat#551221, RRID: AB_394099), anti-human IFN-γ antibody (BD Biosciences Cat#555051, RRID: AB_395672) or anti-mouse IFN-γ (AN-18) antibody (Thermo Fisher Scientific Cat#14-7313-81; RRID:AB_468471) washed, blocked, and incubated with 50μL of assay supernatant at 4°C overnight. PBS dilution of supernatants was applied where necessary. Samples were then discarded and biotin-conjugated anti-human IFN-gamma (BD Biosciences Cat#554550, RRID: AB_395472), anti-human IL-2 (BD Biosciences Cat#555040, RRID: AB_395666) or anti-mouse IFN-γ (BD Pharmingen Cat#551506; RRID:AB_394224) primary detection antibodies and secondary Streptavidin-POD conjugate (Roche Cat#11089153001) incubated successively following 4 PBS-Tween (0.05%) washing steps between each step. Detection was performed with TMB substrate solution (Life technologies) incubated for 15–30 min in the dark and stopped with 500mM sulfuric acid.

Concentration of secreted Interleukin-6 in assay supernatants was determined utilizing the ELISA MAX Deluxe Set Human IL-6 (BioLegend Cat#430504) according to the manufacturer’s manual.

Assay ODs were measured using the Multiscan Go ELISA Reader (ThermoFisher) and concentrations calculated from standard curves using Imukin (Boehringer Ingelheim Cat#72355-01) and Proleukin S (Novartis PZN 2238131) where applicable.

#### Differentiation of mesenchymal stem cells

Mesenchymal stem cells were cultured in Mesenchymal Stem Cell Growth Medium 2. To achieve differentiation, cells were treated with varying concentrations of recombinant TGF-beta 3 (10–20 ng mL^−1^; Figure S4B). Cells were then characterized via flow cytometry analysis with anti-CD19 APC-linked (ImmunoTools Cat#21270196) as well as anti-CD248 FITC-linked (Bioss Cat#bs-2101R-FITC) antibodies. MACS isolation was achieved using the same antibodies combined with anti-APC MicroBeads (Miltenyi Biotec #130-090-855, RRID: AB_244367) on the autoMACS Pro (Miltenyi Biotec, Bergisch Gladbach, Germany) platform using the POSSEL (positive selection standard mode) separation program.

#### Characterization of cell lines and primary cells via FC

Human tumor cell line expression of antigens and receptors was characterized using FACS analysis utilizing the Canto II (Becton Dickinson, Franklin Lakes, USA) platform. Cells were quantified and isolated from culture at 5x10^5^-1x10^6^ cells per FACS tube, stained with anti-CD19 FITC (Miltenyi Biotec Cat#130-113-645; RRID:AB_2726198), anti-CD80 BV421 (BioLegend Cat#305222; RRID:AB_2564407) or anti-CD80 PE (ImmunoTools Cat#21270804; RRID:AB_2923118) and anti-CD86 APC (ImmunoTools Cat#21480866; RRID:AB_2923116), washed twice each before and after antibody application with PBS and incubated at 4°C for 30 min before analysis.

Murine tumor cell line and primary cell expression of antigens was evaluated on the MACSQuant X (Miltenyi Biotec, Bergisch Gladbach, Germany) and Canto II (Becton Dickinson, Franklin Lakes, USA) platforms using anti-mouse CD19 (1D3) APC (ImmunoTools Cat#22270196X2; RRID requested) or anti-mouse CD19 BV510 (6D5) (BioLegend Cat#115545; RRID:AB_2562136), anti-mouse CD80 (16-10A1) PE (BioLegend Cat#104707; RRID:AB_313128) and anti-mouse CD86 (GL-1) PE/Cy7 (BioLegend Cat#105014; RRID:AB_439783) antibodies.

#### Immuno-histological analyses

Immuno-histological studies of human tissue derived from DLBCL patients (before and after CAR T cell treatment or in relapse) were performed on 4-μm-thick sections of the formalin-fixed paraffin-embedded tumor tissue biopsies in whole-section form (University Cologne, Institute for Pathology). For human CD80 detection, slides were primary stained with biotin-labelled polyclonal antibody (5.0 μg mL^−1^) against CD80 (Thermo Fisher Scientific Cat#13-0809-82, RRID:AB_466513) and secondary with streptavidin-POD (500 mU mL^−1^) (Roche Diagnostics Cat#11089153001). For human CD86 detection, slides were primary stained with the mouse monoclonal conjugated CD86-specific antibody (2.0 μg mL^−1^), clone SPM600 (Novus Biologicals Cat#NBP2-44515, RRID:AB_2923113) and secondary with polyclonal HRP-conjugated mouse IgG1-specific antibody (2.0 μg mL^−1^). Subsequently, both CD80 and CD86 stained sections were additionally incubated with DAB chromogen substrate (Vector Laboratories) and with Hematoxylin (PanReac AppliChem) for immune-histological analyses according to manufacturer’s instructions. Representative optical fields were recorded using the Olympus UC90 4K microscope (Olympus, Tokyo, Japan) and processed using ImageJ version 1.53 (National Institutes of Health).^61^ Quantitative assessment of antigen expression and tumor and tumor microenvironment cell classification was performed by a pathologist evaluating 5 high-power fields per slide.

Immunohistological studies of transplanted human tumors derived from mice after CAR T cell treatment were performed on sections of cryo-embedded tumor biopsies. Tumor tissue was embedded in Tissue-Tek O.C.T. Compound (Sakura Finetek Europe B.V.), and 5-μm cryostat sections were fixed in ice-cold acetone. Tissue sections were stained for the presence of CAR T cells using fluorochrome-conjugated antibodies specific for human CD3 AF532, clone UCHT1 (Thermo Fisher Scientific Cat#58-0038-42, RRID:AB_11218675) and Biotin-labelled CD19-CAR antibody, clone REA1298 (Miltenyi Cat#130-127-349, RRID:AB_2923109) and Streptavidin-AF488 (BioLegend Cat#405235) and for the human B cell antigen expression on tumor cells by using AF647-labelled anti human CD19 antibody, clone HIB19 (BioLegend Cat# 302220, RRID:AB_389335), Biotin-labelled anti-human CD80, clone 2D10.4 (ThermoFisher Cat#13-0809-82, RRID:AB_466513) and Streptavidin-AF488 (BioLegend Cat#405235) and AF532-labelled human CD86-specific antibody, clone BU63 (Novus Biologicals Cat#NBP2-34569AF532, RRID:AB_2923133). Slides were analyzed using an Olympus FV 1000 microscope (Olympus, Tokyo, Japan).

CD19 detection in slides from spleens of mice treated in the second-line xenograft trial was performed with a mouse anti-human CD19 (BioLegend Cat#302202; RRID:AB_314232), Biotin labeled anti-mouse IgG1 (BioLegend Cat#406603; RRID:AB_315062), Streptavidin-HRP and DAB chromogen (Vector Laboratories). Cells were also stained with Hematoxylin (PanReac AppliChem). Slides were analyzed with an Olympus BX53 microscope (Olympus, Tokyo, Japan).

#### Mitochondrial gene expression analysis via qRT-PCR

CAR (2^nd^ Gen) and CAR/CCR T cells were stimulated with irradiated (30Gy) SU-DHL-10 cells and kept in culture for 7 and 14 days, with media changed every 2 days and supplemented with 30U mL^−1^ IL-2, 10 ng mL^−1^ IL-7 and 10 ng mL^−1^ IL-15. On the last day of the culture, cells were extracted from assay wells, washed in cold PBS, and analyzed.

To analyze gene expression, total RNA was extracted by the RNeasy Mini Kit (Qiagen, Hilden, Germany) and after DNase digestion (Qiagen) reverse transcribed into complementary DNA (cDNA) using SuperScript III Reverse Transcription kit (Thermo Fisher Scientific) according to the manufacturer’s instruction. cDNA samples were analyzed by LightCycler qPCR (Roche Diagnostics, Mannheim, Germany) using the human hydroxymethylbilane synthase (HMBS) as a reference gene within the Relative Quantification Software (Roche Diagnostics). Primer sequences: MTCO1fw: 5′-TTAGCTGACTCGCCAC-3‘, MTCO1rev: 5‘-GTAACGTCGGGGCATT-3‘, TFAMfw: 5‘-CCAAGAAGCTAAGGGTG-3‘, TFAMrev: 5‘-TTGTGCGACGTAGAAG-3‘, NRF1fw: 5‘-CCACACATAGTATAGCTCATCT-3‘, NRF1rev: 5‘-TTTGTTCCACCTCTCCAT-3‘, NRF2fw: 5‘-TAGTGCGAAAGCAGCC-3‘, RF2rev: 5‘-TTTACGCTGTCCCCAT-3‘, HMBSfw: 5‘-TGCACGATCCCGAGAC-3‘, HMBSrev: 5‘-CGTGGAATGTTACGAGC-3‘.

#### Western Blot analysis of 4-1BB signaling

1.3x10^6^ CAR/CCR and CAR (2^nd^ Gen) T cells were cultured either with 1.3x10^6^ SU-DHL-10 cells, SU-DHL-10 cells and 20 μg mL^−1^ Ipilimumab or by themselves for 24 h. They were then put on ice, washed in PBS at 4°C and lysed with 500μL RIPA buffer with protease and phosphatase inhibitors. Protein concentration was determined with the Pierce BCA Protein Assay Kit (Thermo Scientific Cat#23227) according to the manufacturer’s instructions and 0.5μg of protein per sample loaded into a NuPage Bis-Tris 17 well pre-cast gel (Invitrogen Cat#NP0329) with reducing agent, loading buffer and deionized water for a total volume of 15μL. The gel was run at 200V until optimal ladder separation, blotted, blocked and stained with anti-*p*-IKK alpha/beta (S176 + S180) (Bioss Cat#bs-3237R; RRID:AB_10883648) and anti-Rabbit IgG HRP (Santa Cruz Biotechnology Cat#sc-2030; RRID:AB_631747) before being developed with Pierce ECL Western Blotting Substrate (Thermo Scientific Cat#32106). Chemiluminescence was recorded with the Vilber Fusion Solo S (Vilber, Collégien, France) and merged with the ladder in the accompanying software.

### Quantification and statistical analysis

Where not otherwise noted, raw data was analyzed and visualized using R within the RStudio IDE augmented by the packages ggpubr, survival, survminer, and rstatix. Figures 1C and 6D were created with Graphpad Prism and Microsoft Excel respectively.

Plots report the arithmetic mean with error bars reporting standard error of the mean where applicable. Where not otherwise noted, statistical significance was evaluated using a two-sided unpaired non-adjusted t-test with significance level alpha = 0.05 (5%). Significance levels were reported according to p values (∗ ≤0.05; ∗∗ ≤0.01; ∗∗∗ ≤0.001; ∗∗∗∗ ≤0.0001). Statistical significance in transcriptomic data was evaluated using a one-sided unpaired non-adjusted Mann-Whitney-U test with significance levels reported according to p values (∗ ≤0.05; ∗∗ ≤0.01; ∗∗∗ ≤0.001; ∗∗∗∗ ≤0.0001). Other statistical parameters (e.g., n) are reported in the figure legends, or in method details for *in vivo* data.

Graphical representations of assay procedures and receptor and vector designs as well as the graphical abstract were created and individually licensed with BioRender.com.

## Supplemental information

Document S1. Figures S1‒S5

Document S2. Article plus supplemental information
