# Supplementary material for: An anti-CD19/CTLA-4 switch improves efficacy and selectivity of CAR T cells targeting CD80/86-upregulated DLBCL
Source: Cell Rep Med. 2024 Feb 9;5(2):101421. doi: 10.1016/j.xcrm.2024.101421 (PMC10897622; doi:10.1016/j.xcrm.2024.101421)
Supplement: Document S1. Figures S1‒S5 [file mmc1.pdf]

Cell Reports Medicine, Volume *5*

Supplemental information

An anti-CD19/CTLA-4 switch improves efficacy and selectivity of CAR T cells targeting CD80/86-upregulated DLBCL

Lars Fabian Prinz, Tobias Riet, Daniel Felix Neureuther, Simon Lennartz, Danuta Chrobok, Hanna Hübbe, Gregor Uhl, Nicole Riet, Petra Hofmann, Marianna Hösel, Adrian Georg Simon, Luis Tetenborg, Paul Segbers, Joji Shimono, Philipp Gödel, Hyatt Balke-Want, Ruth Flümann, Gero Knittel, Hans Christian Reinhardt, Christoph Scheid, Reinhard Büttner, Björn Chapuy, Roland Tillmann Ullrich, Michael Hallek, and Markus Martin Chmielewski

A

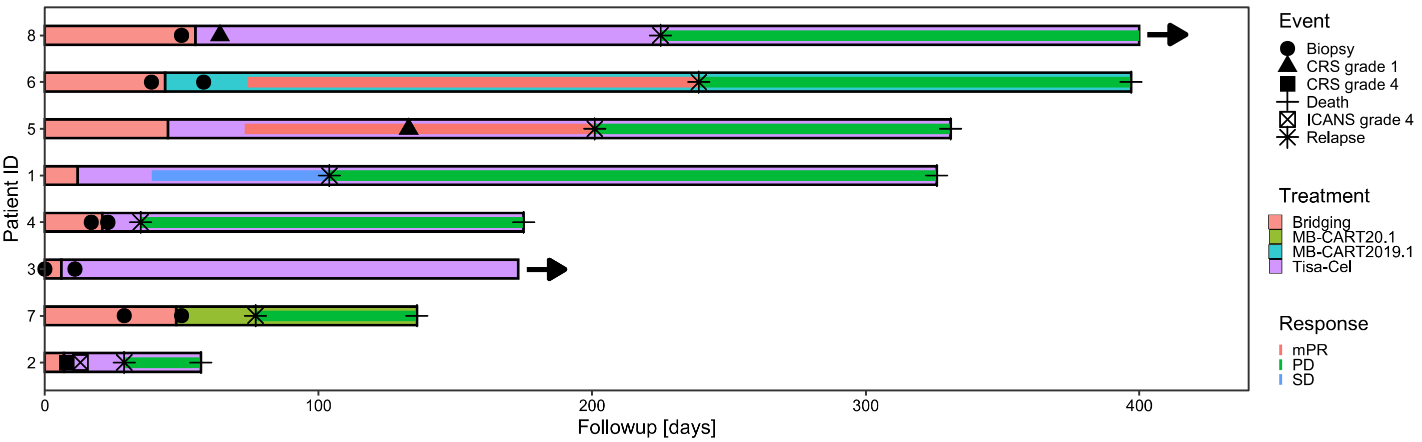

B

Patient	1	2	3	4	5	6	7	8
Age	55	38	67	72	67	77	60	48
Sex assigned at birth	M	F	M	F	M	F	M	M
ECOG	0	1	0	1	1	1	1	0
Entity	DLBCL	DLBCL	DLBCL	DLBCL	DLBCL	tFL	DLBCL	FL
COO	ABC	GCB	GCB	UC	GCB	GCB	GCB	n/a
DH/TH		DH			DH			
ki67 %	100	90		80	90	10	60	25
IPI	2	0	4	4	3	1	1	2
Stage	4	2	4	4	4	2	4	4
Bulk	-	-	-	-	-	-	-	-
Extranodal lesion	+	+	-	+	+	-	+	+
Relapse	+	-	-	+	+	+	+	+

C

Patient	1	2	3	4	5	6	7	8
ICU	-	+	-	-	-	-	-	-
IMC	-	+	-	-	-	-	+	-
CRS (Grade)	-	4	-	-	1	-	-	1
ICANS (Grade)	-	4	-	-	-	-	-	-
Infection	-	+	-	-	-	-	-	-
Drugs/Blood products								
Tocilizumab	-	+	-	-	-	-	-	-
Steroids	-	+	-	-	-	-	-	-
G-CSF	-	-	-	-	-	-	-	+
Platelets	-	+	-	-	+	-	-	+
Cytopenias								
Neutropenia <1000/µl	+	+	-	+	-	-	+	+
Neutropenia <500/µl	-	+	-	-	-	-	+	+
Anemia grade 3/4	-	+	-	-	+	-	-	-
B cell aplasia	+	+	-	-	+	+	+	+
IgG <5 g/L	+	+	-	-	+	+	+	+

D

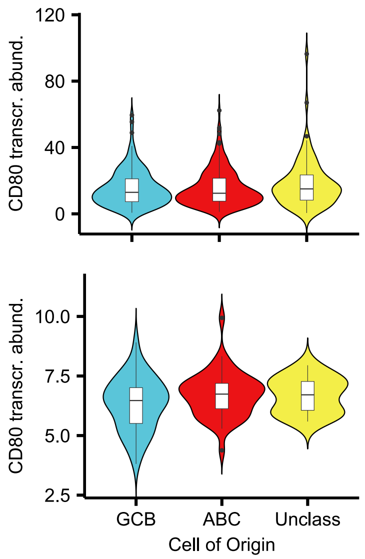

Figure S1, related to Fig 1: Clinical and demographic data of IHC sample patients and CD80 expression plots.

A-C Clinical courses, patient, and tumor characteristics of CAR T patients in IHC analyses. **D** CD80 transcriptional abundance across COOs in Schmitz set (upper plot) and Chapuy set (lower plot).

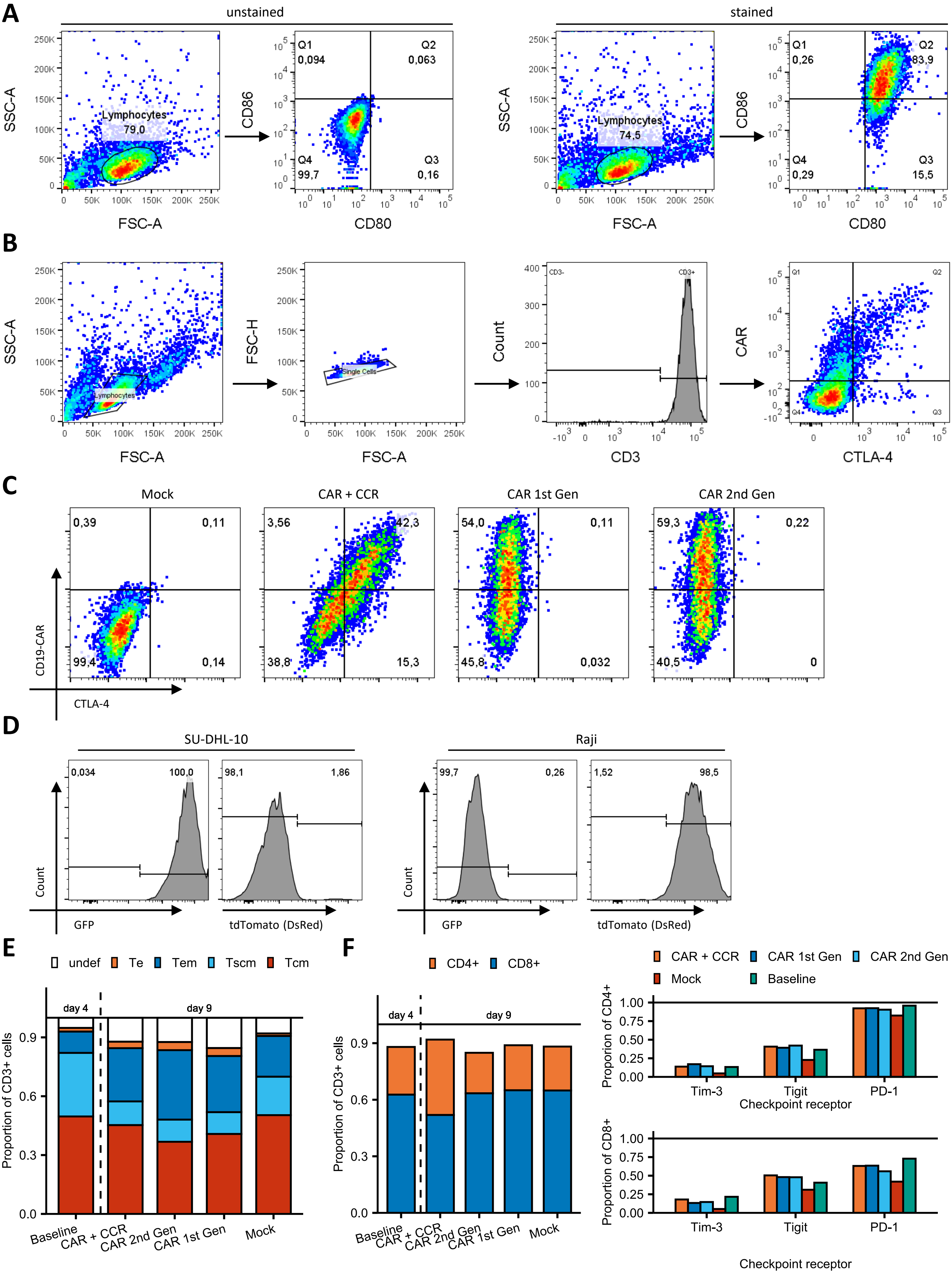
A
unstained
stained
SSC-A
FSC-A
Lymphocytes
79,0
74,5
CD86
CD80
Q1
0,094
Q2
0,063
Q4
99,7
Q3
0,16
Q1
0,26
Q2
83,9
Q4
0,29
Q3
15,5
B
SSC-A
FSC-A
Lymphocytes
FSC-H
Single Cells
Count
CD3
CD3-
CD3+
CAR
CTLA-4
Q1
Q2
Q3
Q4
C
Mock
CAR + CCR
CAR 1st Gen
CAR 2nd Gen
0,39
0,11
99,4
0,14
3,56
42,3
38,8
15,3
54,0
0,11
45,8
0,032
59,3
0,22
40,5
0
CD19-CAR
CTLA-4
D
SU-DHL-10
Raji
0,034
100,0
98,1
1,86
99,7
0,26
1,52
98,5
Count
GFP
tdTomato (DsRed)
E
undef
Te
Tem
Tscm
Tcm
day 4
day 9
Proportion of CD3+ cells
Baseline
CAR + CCR
CAR 2nd Gen
CAR 1st Gen
Mock
F
CD4+
CD8+
day 4
day 9
Proportion of CD3+ cells
Baseline
CAR + CCR
CAR 2nd Gen
CAR 1st Gen
Mock
CAR + CCR
CAR 1st Gen
CAR 2nd Gen
Mock
Baseline
Proporion of CD4+
Tim-3
Tigit
PD-1
Checkpoint receptor
Proportion of CD8+
Tim-3
Tigit
PD-1
Checkpoint receptor

Figure S2, related to Fig 2: Gating strategies and additional plots related to Figure 2 and expression of fluorochromes in lymphoma cell lines

A Flow cytometry gating strategy used to obtain CD80/CD86 expression in lymphoma tumor cell lines (Figure 2E) following the example of the Raji cell line. **B** Flow cytometry gating strategy used to obtain flow cytometry plots showing expression of CAR and CCR receptors on primary healthy donor T cells (Figure 2C). **C** CD19 CAR and CTLA-4 CCR expression of transfected HEK293t cells to eliminate possible endogenous CTLA-4 expression on T cells. (Figure 2C) **D** Fluorochrome expression in lymphoma tumor cell lines used for in vitro cytotoxicity assays (Figure 3A) **E** Phenotype of T cells according to their differentiation into effector (Te), effector memory (Tem), central memory (Tcm) and stem cell-like central memory (Tscm) T cells before (day 4) and after (day 9) transduction. **F** Phenotype of T cells according to their expression of CD4 and CD8 and their checkpoint receptor expression.

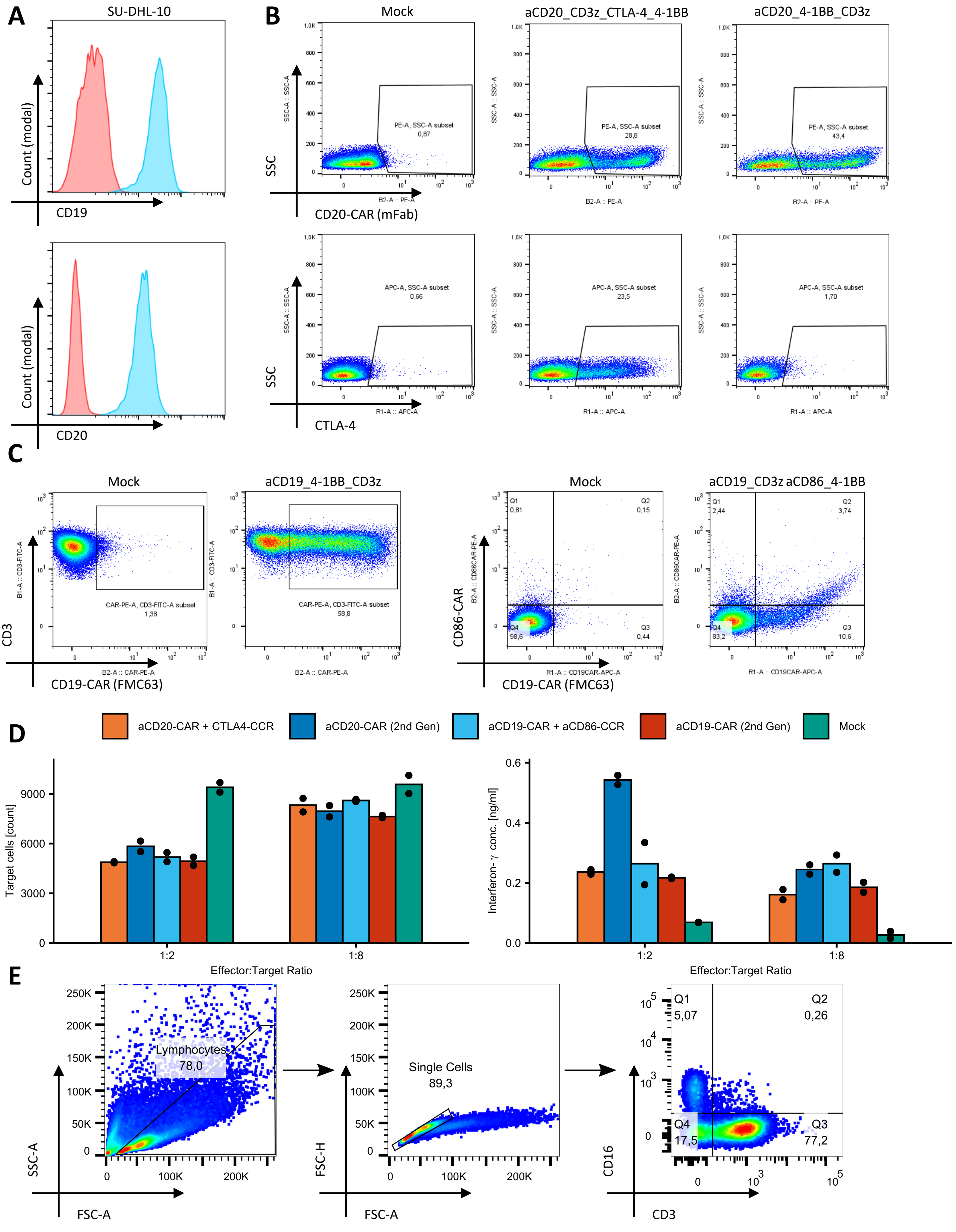

A
SU-DHL-10
Count (modal)
CD19
Count (modal)
CD20
B
Mock
aCD20_CD3z_CTLA-4_4-1BB
aCD20_4-1BB_CD3z
SSC
CD20-CAR (mFab)
PE-A, SSC-A subset
0,87
28,8
43,4
SSC-A :: SSC-A
B2-A :: PE-A
SSC
CTLA-4
APC-A, SSC-A subset
0,66
23,5
1,70
R1-A :: APC-A
C
Mock
aCD19_4-1BB_CD3z
CD3
CD19-CAR (FMC63)
B1-A :: CD3-FITC-A
B2-A :: CAR-PE-A
CAR-PE-A, CD3-FITC-A subset
1,38
58,8
Mock
aCD19_CD3z aCD86_4-1BB
CD86-CAR
CD19-CAR (FMC63)
B2-A :: CD86CAR-PE-A
R1-A :: CD19CAR-APC-A
Q1 0,81
Q2 0,15
Q3 0,44
Q4 98,6
Q1 2,44
Q2 3,74
Q3 10,6
Q4 83,2
D
aCD20-CAR + CTLA4-CCR
aCD20-CAR (2nd Gen)
aCD19-CAR + aCD86-CCR
aCD19-CAR (2nd Gen)
Mock
Target cells [count]
Effector:Target Ratio
1:2
1:8
Interferon-γ conc. [ng/ml]
Effector:Target Ratio
1:2
1:8
E
SSC-A
FSC-A
Lymphocytes
78,0
FSC-H
FSC-A
Single Cells
89,3
CD16
CD3
Q1 5,07
Q2 0,26
Q3 77,2
Q4 17,5

Figure S3, related to Fig 2: The CAR + CCR concept works even with its binding domains exchanged.

A CD19 and CD20 expression of SU-DHL-10 cell line (blue) compared to isotype control (red). **B** Expression of aCD20CAR and aCTLA4CCR on T cells. **C** Expression of aCD19CAR and CD3 on CAR (2nd Gen) T cells and of aCD19CAR and aCD86CCR on CAR/CD86CCR T cells. **D** Killing of CD19+CD20+ SU-DHL-10 cells and associated Interferon-γ secretion by aCD20CAR(/CCR) and aCD19CAR/CD86CCR T cells compared with CAR (2nd Gen) and Mock.

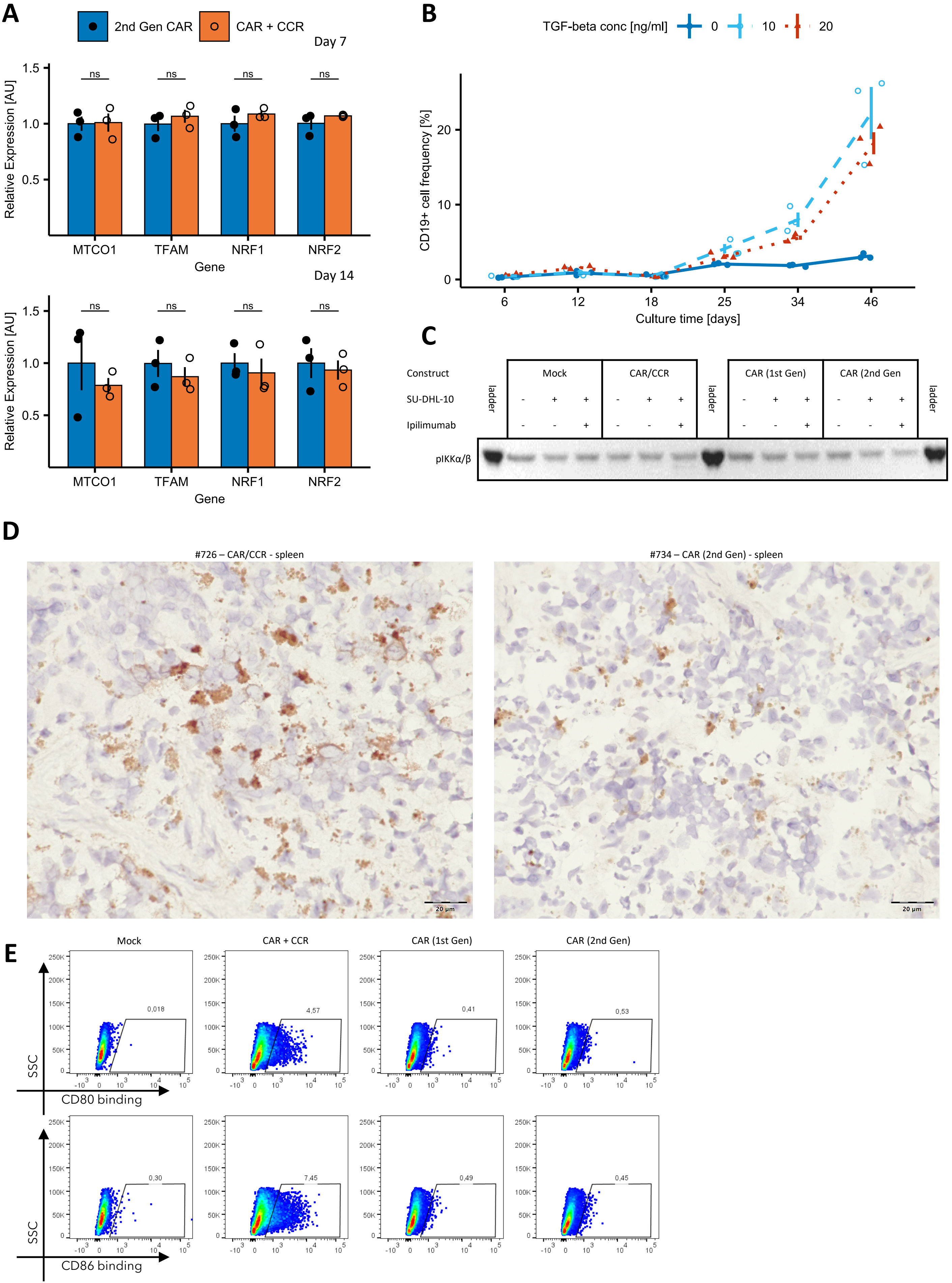
A
2nd Gen CAR
CAR + CCR
Day 7
Relative Expression [AU]
1.5
1.0
0.5
ns
MTCO1
TFAM
NRF1
NRF2
Gene
Day 14
B
TGF-beta conc [ng/ml]
0
10
20
CD19+ cell frequency [%]
Culture time [days]
6
12
18
25
34
46
C
Construct
Mock
CAR/CCR
CAR (1st Gen)
CAR (2nd Gen
SU-DHL-10
Ipilimumab
ladder
pIKKα/β
D
#726 – CAR/CCR - spleen
#734 – CAR (2nd Gen) - spleen
20 µm
E
Mock
CAR + CCR
CAR (1st Gen)
CAR (2nd Gen)
SSC
CD80 binding
CD86 binding
0,018
4,57
0,41
0,53
0,30
7,45
0,49
0,45

Figure S4, related to Fig 3, 5, 6: Additional data on CAR/CCR T cell molecular biology, pericyte differentiation, target persistence in treated mice and target antigen binding by the CCR

A Results of qRT-PCR of mitochondrial genes and pro-mitochondrial transcription factors comparing CAR (2nd Gen) and CAR/CCR over 7 and 14 days. **B** Time series of CD19 expression on VW-MSC derived pericytes. (see Figure 5C-E) **C** Western blot stained for phospho-IKKa/b with each lane corresponding to a construct either without target cells, with target cells but without ipilimumab or with both target cells and ipilimumab. (Stained lane at ~64 kD is shown with the rest of the gel only showing ladder staining removed) **D** Representative pictures of spleen slides of mice from second-line xenograft mouse trial (see Figure 5) stained to show human-CD19 expression with DAB chromogen. **E** Binding of CD80 and CD86 on transfected 293t cells detected with recombinant Fc-linked CD80 and CD86 and a FITC-linked anti-human IgG antibody.

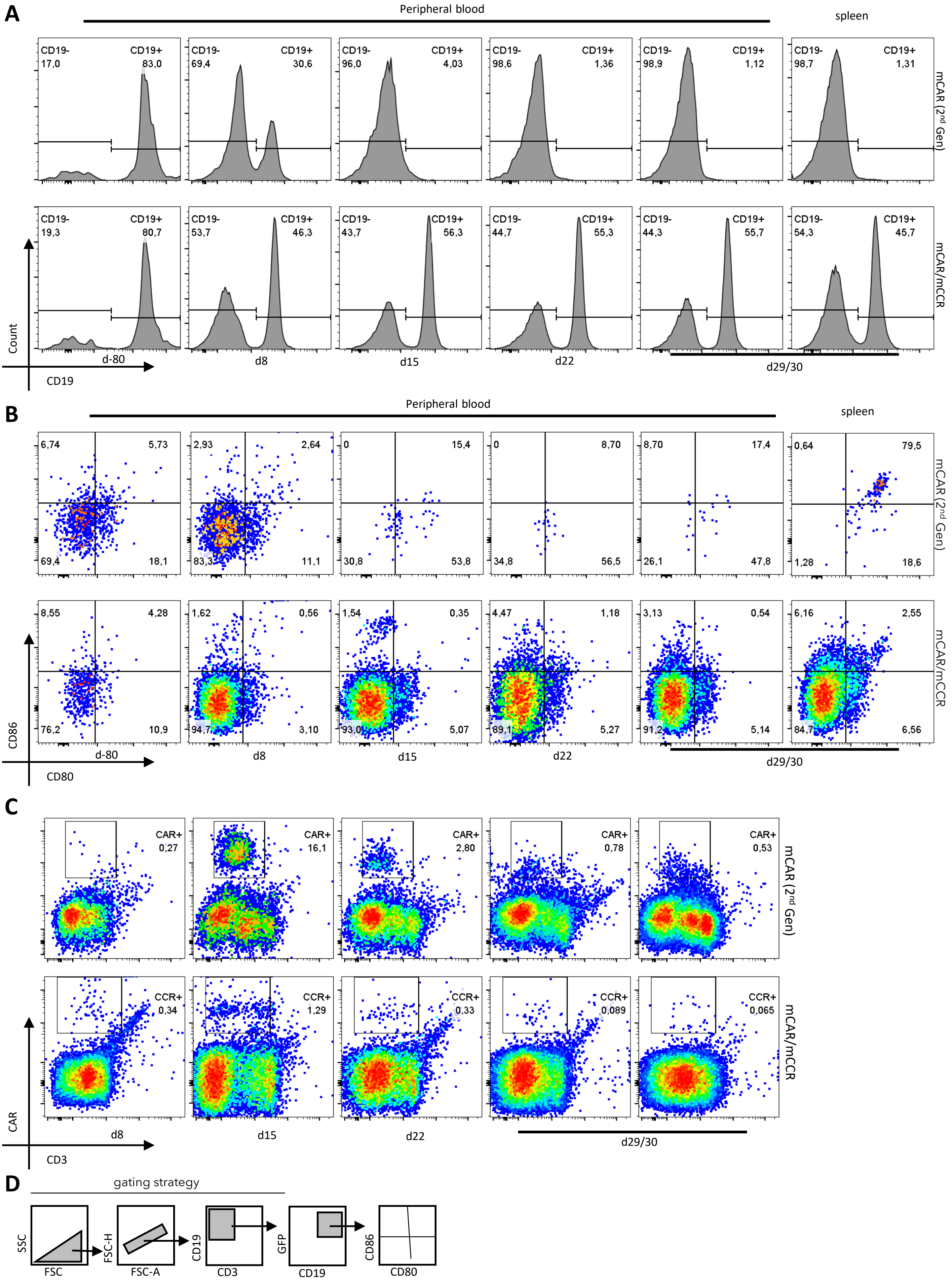
A
Peripheral blood
spleen
CD19-
CD19+
17,0
83,0
69,4
30,6
96,0
4,03
98,6
1,36
98,9
1,12
98,7
1,31
mCAR (2nd Gen)
19,3
80,7
53,7
46,3
43,7
56,3
44,7
55,3
44,3
55,7
54,3
45,7
mCAR/mCCR
Count
CD19
d-80
d8
d15
d22
d29/30
B
Peripheral blood
spleen
6,74
5,73
69,4
18,1
2,93
2,64
83,3
11,1
0
15,4
30,8
53,8
0
8,70
34,8
56,5
8,70
17,4
26,1
47,8
0,64
79,5
1,28
18,6
mCAR (2nd Gen)
8,55
4,28
76,2
10,9
1,62
0,56
94,7
3,10
1,54
0,35
93,0
5,07
4,47
1,18
89,1
5,27
3,13
0,54
91,2
5,14
6,16
2,55
84,7
6,56
mCAR/mCCR
CD86
CD80
d-80
d8
d15
d22
d29/30
C
CAR+
0,27
16,1
2,80
0,78
0,53
mCAR (2nd Gen)
CCR+
0,34
1,29
0,33
0,089
0,065
mCAR/mCCR
CAR
CD3
d8
d15
d22
d29/30
D
gating strategy
SSC
FSC
FSC-H
FSC-A
CD19
CD3
GFP
CD19
CD86
CD80

Figure S5, related to Fig 6: Gating strategy for Figure 6D and results of B cell depletion in C57BL/6J mice.

A Proportion of CD19+ B cells over time in C57BL/6J mice injected with mCAR and mCAR/mCCR. **B** CD80 and CD86 expression of CD19+ B cells in Supplemental Figure 5A. **C** mCAR and mCAR/mCCR cells over time in peripheral blood and spleen samples of C57BL/6J mice. **D** Gating strategy used to evaluate CD80/86 positivity rate in in vivo selectivity trial (see Figure 6D).
